# Spiritual Care in Palliative Care: A Systematic Review of the Recent European Literature

**DOI:** 10.3390/medsci7020025

**Published:** 2019-02-07

**Authors:** Marie-José H. E. Gijsberts, Anke I. Liefbroer, René Otten, Erik Olsman

**Affiliations:** 1 End-of-Life Care Research Group, Vrije Universiteit Brussel & Ghent University, Laarbeeklaan 103, 1090 Brussel, Belgium; 2 Faculty of Religion and Theology, Vrije Universiteit Amsterdam, De Boelelaan 1115, 1081 HV Amsterdam, The Netherlands; a.i.liefbroer@vu.nl; 3 VU University Library, Vrije Universiteit Amsterdam, De Boelelaan 1117, 1081 HV Amsterdam, The Netherlands; r.otten@vu.nl; 4 Department of Medical Ethics & Health Law, Leiden University Medical Center, 2333 ZA Leiden, The Netherlands; erik.olsman@lumc.nl; 5 Department of Spiritual Care, Hospice Bardo, 2131 BM Hoofddorp, The Netherlands

**Keywords:** palliative care, hospice, end of life, spirituality, spiritual care, meaning, religion

## Abstract

Many studies on spiritual care in palliative care are performed in the US, leaving other continents unexplored. The objective of this systematic review is to map the recent studies on spiritual care in palliative care in Europe. PubMed, CINAHL, ATLA, PsycINFO, ERIC, IBSS, Web of Science, EMBASE, and other databases were searched. Included were European studies published in a peer-reviewed journal in 2015, 2016, or 2017. The characteristics of the included studies were analyzed and a narrative synthesis of the extracted data was performed. 53 articles were included. Spiritual care was seen as attention for spirituality, presence, empowerment, and bringing peace. It implied creative, narrative, and ritual work. Though several studies reported positive effects of spiritual care, like the easing of discomfort, the evidence for spiritual care is low. Requirements for implementation of spiritual care in (palliative) care were: Developing spiritual competency, including self-reflection, and visibility of spirituality and spiritual care, which are required from spiritual counselors that they participated in existing organizational structures. This study has provided insight into spiritual care in palliative care in Europe. Future studies are necessary to develop appropriate patient outcomes and to investigate the effects of spiritual care more fully.

## 1. Introduction

Spiritual care is an intrinsic and essential component of palliative care, central to Cicely Saunders’ understanding [1], and recognized by and included in the World Health Organization definition of palliative care for almost 15 years [2,3]. There is growing evidence that spiritual care at the end of life is important to patients and that patients want health care professionals to provide this type of care [4,5]. The positive effects of spiritual care on patients’ quality of life have been reported across age groups and patient groups/medical conditions, including cancer, organ failure, and dementia [6,7,8,9,10,11,12,13,14]. There is also evidence that lack of spiritual support by health care teams is associated with poor quality of life, dissatisfaction with care, less hospice utilization, more aggressive treatment, and increased costs, particularly among ethnic minority groups and patients with high levels of religious coping [6,15,16,17]. Despite this emerging evidence and its status as a core dimension of palliative care, spiritual care remains the least developed and most neglected dimension of palliative care [18,19,20,21].

Consequently, in recent years, initiatives have been established to promote the integration of this dimension of palliative care in research and clinical care. To start with clinical care, we should mention that in 2010, members of the European Association for Palliative Care (EAPC) founded a Spiritual Care Taskforce, which aims to “further evidence-based spiritual care by developing an agenda to inform research in this area, to improve staff competence and confidence and outcomes for patients and carers” [22]. Progress is also evident at national levels. In the UK, for example, National Health Service (NHS) Scotland has led on developing information and training materials for spiritual care for all NHS health care professionals [23]. In Germany, the International Society for Health and Spirituality (IGGS) was founded in 2011, aiming to develop an understanding of spirituality and spiritual care among health care professionals in German-speaking countries [24]. The Global Network for Spirituality and Health (GNSAH) was formed in the USA in 2013, with one of the explicit goals being to build “the knowledge and evidence base related to spirituality and health” [25].

Since 2005, several review studies have been conducted in order to describe research on spiritual care in palliative care. A thematic review of literature published between 1980 and 2005 recommended more rigorous qualitative research, both within palliative care and spirituality and health literature in general [26]. In 2010, Holloway et al. conducted a systematic review of the English written research literature published between 2000 and 2010. Most of their included studies had been conducted in the US, where ‘spiritual’ is commonly equated with factors which in the UK are more likely to be termed ‘religious’ [27]. In a Cochrane review published in 2012, Candy et al. reported that the five included randomized controlled trails (RCTs) did not show conclusive evidence that interventions with spiritual or religious components enhance spiritual wellbeing [28]. However, in 2016, Kruizinga et al. evaluated the effect of spiritual interventions on the quality of life in patients with cancer, focusing on literature published up to 2014. Based on 12 clinical trials, the researchers found that narrative spiritual interventions can improve the quality of life in cancer patients in the short term, but no evidence was found that this effect was maintained in the long term, up to three–six months [29].

These review studies used different definitions of spiritual care, and consequently, they identified different studies for inclusion. Furthermore, the majority of the included studies had been conducted in the US and most review studies had only included original studies published in English. These limitations require the synthesis of empirical studies on spiritual care in palliative care in other contexts, one of which is Europe, where several languages are spoken and written. Our systematic review aims to map the recent empirical and peer-reviewed studies on spiritual care in palliative care conducted in Europe. More specifically, the objective of this review is to answer the following research questions, with a focus on the recent literature: (1) how is spiritual care in palliative care understood in Europe?; (2) what is the effectiveness of spiritual care in palliative care at this continent?; (3) what is required to implement spiritual care in palliative care in Europe?

This aim and these questions are in line with the research priorities identified by the Spiritual Care Taskforce of the EAPC [30]. They help to understand European perspectives on spiritual care in palliative care. More specifically, they help to understand how research participants in the included studies describe spiritual care and the providers of spiritual care. Therefore, we will not use predefined definitions of spiritual care or spirituality, but use search terms that try to cover as many studies on spiritual care in palliative care as possible. For example, spiritual care includes dimensions of healthcare that are characterized by ‘meaning’ or ‘existential’. Mapping the European perspectives on spiritual care in palliative care, based on empirical studies, is important because it helps to formulate the effects of spiritual care, to identify gaps in the literature, and to describe what is required in future studies.

## 2. Materials and Methods

### 2.1. Literature Search

As palliative care is multidisciplinary in nature and because of the variety of languages spoken and written in Europe, a multi-source search strategy was applied [31,32]. Literature in English, German, Spanish, Portuguese, French and Dutch was searched, and also papers written in other languages were identified, as long as their title or abstract had been written in one of the just mentioned languages. The following databases covering literature on medical, psychological, social, religious and ethical topics were searched: PubMed, CINAHL, ATLA, PsycINFO, ERIC, IBSS, Web of Science, EMBASE, and PiCarta, the joint catalogue of nearly all major Dutch libraries. For literature in German: DIMDI, Ethics in Medicine, Ethmed, PSYNDEX, and MEDPILOT. For literature in Spanish and Portuguese: RCAAP, Bireme, and LILACS.

First, a systematic search strategy within PubMed was conducted. Relevant papers were identified in advance to test the search strategy and see if these papers are picked up or not. If not, the strategy was refined, aiming to include as much of the relevant literature as possible. Initial search terms that appeared to be suitable included: (Spiritual, religion, meaning, pastoral, and faith) and (terminal, end of life, limited life, palliative, hospice, and dying). The search terms were adapted for the other databases, as they use different subject headings on the topics of interest in this study (authors R.O. and M.J.G.). For the search terms of the different databases, see Appendix A. All databases were searched for studies that were published between 1 January 2015 and 31 December 2017, some of which were articles that had been published online first (2015–2017) and published in a journal in 2018.

### 2.2. Inclusion Criteria

Articles were included when they were:

- an empirical study in a European country
- published in a peer-reviewed journal
- concerning spiritual care provided in the context of palliative care
- published between 1 January 2015 and 31 December 2017.
- published full-text in English, German, Spanish, Portuguese, French or Dutch.

Palliative care in this study was defined as the care for individuals living with an incurable, progressive and life-limiting disease or fragile elderly people who are in several countries included in palliative care, and/or the care for family members of these individuals.

### 2.3. Study Selection

**Figure 1:**
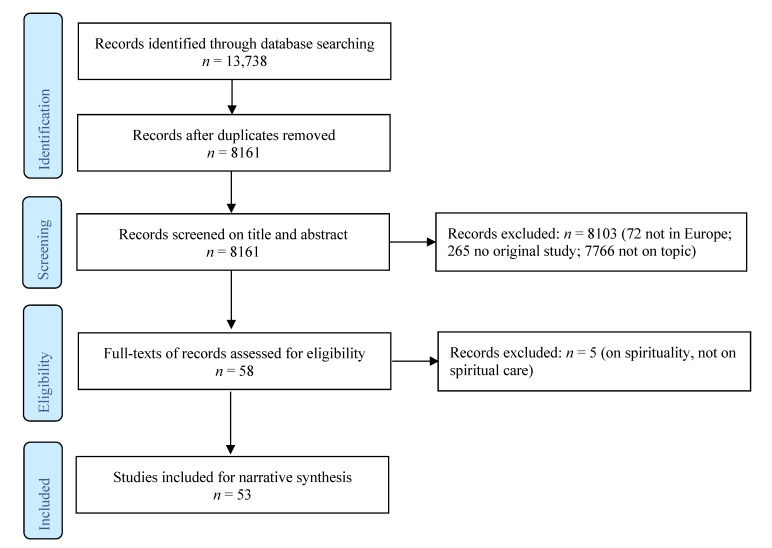
Flowchart.

The primary reviewer (M.J.G.) and the second reviewer (E.O.) independently piloted the selection criteria by assessing the title and abstract of the first 1000 studies to ensure the search strategy was adequate, and to fine-tune the selection criteria. The same procedure was repeated with the primary reviewer (M.J.G.) and the third reviewer (A.I.L.). Then, all three reviewers discussed and determined the final selection criteria until consensus was reached. For details on these criteria, see Appendix B. For the Flowchart, see Figure 1. Subsequently, M.J.G. screened the titles and abstracts of all references. In case of ambiguity on the inclusion or exclusion of a reference, the reference was retained for full-text review, or M.J.G. discussed with E.O., respectively, A.I.L. until they reached consensus. Then, the full texts of the included references were reviewed (M.J.G., E.O., and A.I.L.), and five references were excluded because they reported on spirituality instead of spiritual care. In case of ambiguity, M.J.G. reviewed the full-text and made a final decision.

### 2.4. Data Extraction, Analysis and Synthesis

Data were extracted from the included papers using pre-piloted data extraction tables, designed to meet the objectives of this review. The characteristics of the studies were extracted from the abstracts and full-texts of the papers. Two reviewers (E.O., M.J.G.) extracted the data independently and in the case of ambiguity, they discussed it until they reached consensus. We performed a narrative synthesis of the extraxted data by describing, labelling, grouping, and clustering the data on, for example, methods, study population, palliative setting, and country. For details on the characteristics of the included studies, see Table 1. The extracted data were then analyzed from the research questions (practices, effectiveness, and requirements for implementation) and their definitions of spirituality. For details, see Appendix C. Similarities and differences between these findings were analyzed and discussed by the three reviewers. The preliminary results were discussed by the three reviewers until consensus was reached. 

## 3. Results

### 3.1. Characteristics of the Included Studies

Fifty-three articles were included [33,34,35,36,37,38,39,40,41,42,43,44,45,46,47,48,49,50,51,52,53,54,55,56,57,58,59,60,61,62,63,64,65,66,67,68,69,70,71,72,73,74,75,76,77,78,79,80,81,82,83,84,85]. Seventeen were quantitative studies [33,34,36,37,40,43,50,51,54,61,66,67,69,71,74,81,82], of which the majority (*n* = 11) were survey studies, 27 qualitative studies [38,39,41,42,44,45,46,48,49,53,55,56,57,58,59,60,63,64,68,73,76,78,79,80,83,84,85], most of which were interview studies (*n* = 13), and nine mixed-methods studies [35,47,52,62,65,70,72,75,77]. Most studies were conducted in Western Europe (*n* = 37), especially in the UK (*n* = 10), Germany (*n* = 8), and the Netherlands (*n* = 13). Of the Southern European countries, only several studies conducted in Spain (*n* = 6) were included. A few Northern European studies were found (*n* = 7). Except from one study in the Czech Republic, no studies conducted in Eastern Europe were found (Eurovoc classification). A total of more than thirty thousand participants participated in the studies, with two studies being a substantial part of this number (*n* = 6263 and *n* = 20,907) [33,72]. Most included studies focused on the perspectives of health care professionals, spiritual care receivers, or the interactions between them. For details on the characteristics of the studies, see Table 1.

### 3.2. Practices of Spiritual Care in Palliative Care

In this paragraph, definitions of spirituality and what caregivers do when providing spiritual care are described.

#### 3.2.1. Spirituality

In their introduction section, several included articles referred to the working definition of the Spiritual Care Taskforce of the EAPC: ‘Spirituality is the dynamic dimension of human life that relates to the way persons (individual and community) experience, express and/or seek meaning, purpose and transcendence, and the way they connect to the moment, to self, to others, to nature, to the significant and/or the sacred’ [86]. In a survey, meant to identify spiritual care training courses, 80% of the responding EAPC members used this definition as well [40], and several researchers gave definitions of spirituality which reflected elements of this definition, like meaning or the connection with others or the significant [36,43,58,73,84].

Nevertheless, several authors made their definition more tangible by stating that spirituality is about ‘who I am’, ‘why I am here’ and ‘what can I hope from this moment’ [35], or by referring to themes such as courage and hope [64]. Furthermore, some studies have empirically explored the definitions of spirituality of their participants. For instance, a qualitative study found that the following spiritual issues were relevant for patients with end-stage heart failure and their carers: Love and belonging, hope and coping, meaning and purpose, faith and belief, and existential issues [42]. Health care professionals in another qualitative study described spirituality as searches for meaning and hope. They saw it as a broad, yet highly individualized construct, which might include orthodox as well as unorthodox beliefs [38].

Though definitions of spirituality often contain elements like (the search for) meaning, relationships and hope, a single definition that covers all dimensions of spirituality is lacking [45]. However, a good understanding of spirituality and spiritual care was required to implement spiritual care in palliative care in several health care settings, like hospice care in a German study [84], or palliative care consultation teams in the Netherlands [36]. A clustered RCT concluded that more research is needed to better understand the constructs of spirituality that are relevant for the provision of spiritual care by Belgian general practitioners [67].

#### 3.2.2. Spiritual Care

Participants in several included studies referred to the importance of ‘being there’ or presence in spiritual care. For example, people with mild intellectual disabilities in a qualitative study expressed the value of ‘being there’ in a spiritual sense [49], and nurses, psychologists, and spiritual caregivers in a qualitative study in the UK exemplified this as follows: They listened and bore witness to patients’ suffering [38]. ‘Being there’ implied that the spiritual caregiver recognized the shared humanity of each person [41].

While ‘being there’, spiritual caregivers had to pay attention to the spirituality of their patients. A controlled trial in the Netherlands, for example, reported that patients found attention to their spiritual needs very important [81], and a Spanish study described that caregivers should identify spiritual comments made by patients [61]. Another Spanish study developed a tool that helped to pay attention to psychosocial and spiritual needs of patients and their families [35]. Paying attention to spirituality included the examination of patients’ and families’ hope. Patients with end-stage heart failure in one study, for instance, mentioned the importance of keeping their hopes alive [42].

Another study described a relationship between hope, empowerment and compassion [59], and physicians in a German study used methods to empower their oncology patients [55]. Spiritual caregivers in other studies brought peace or tried to bring peace to their patients. A qualitative study with Norwegian hospital nurses, for instance, reported that participants tried to help their patients to find peace [46], and nurses and volunteers in another qualitative study saw their spiritual care as successful when they were able to mitigate patients’ (existential) fears [84]. Chaplains in Dutch focus groups described several nursing interventions of which one was to create a peaceful environment, for instance by playing music and dimming light [58].

According to several studies, spiritual care was an art, which itself utilized the arts, like the visual or auditive arts. Object elicitation of patients with advanced cancer in a phenomenological study, for example, facilitated the articulation and expression of experiences [68]. Other studies described music therapy [62] or dance movement therapy [48], in which there was an overlap between psychosocial and spiritual care. Spiritual care as an art also included the use of narrative art, like the spiritual history taking in a German qualitative study [78], and the spiritual reminiscence in a Finnish study [80]. Other studies reported on the art of performing rituals [38,63].

We summarize this paragraph as follows. The working definition of the EAPC is used frequently and other definitions often included elements such as (the search for) meaning and significant relationships. Good understanding of spirituality is required to implement spiritual care in palliative care. In addition, participants in the included studies tried to be present, while paying attention to the spirituality of their patients and patients’ family members, including their hopes. They empowered them and tried to bring peace to their patients and patients’ relatives. In so doing, spiritual care was an art, which included not only the use of visual and auditive arts, but also narrative and ritual work.

### 3.3. Effectiveness of Spiritual Care in Palliative Care

In this paragraph, the effectiveness of spiritual care based on two RCTs is described. It also describes the benefits of spiritual care based on other studies. 

#### 3.3.1. Randomized Controlled Trials

Vermandere et al. investigated the effect of taking a spiritual history based on the ars moriendi model with patients in palliative home care in Belgium. Based on a survey and semi-structured interviews with nurses and physicians (*n* = 24), the researchers found that this spiritual assessment was perceived as valuable because patients had been able to share their expectations and wishes about the end of life. Participants felt that the relationship with their patient had been strengthened [47]. However, the clustered RCT conducted to quantitatively measure the effect of spiritual history taking on patients’ spiritual well-being, quality of life, pain, and patient–provider trust (49 dyads) yielded no significant differences between the intervention and control group [67].

Kögler et al. conducted an RCT with relatives of palliative care patients (*n* = 130) in Germany. The aim was to investigate the relation between mindfulness, mental distress, and psychological well-being and to investigate the effect of Existential Behavioral Therapy (EBT) on mindfulness, consisting of mindfulness training as a central part. Mindfulness in itself correlated negatively with mental distress and positively with life satisfaction. The intervention had a small but significant effect on mindfulness as a state, and the authors therefore concluded mindfulness to be promising in supporting informal caregivers of palliative care patients [37].

#### 3.3.2. Other Studies

Other studies have explored the effects, potential benefits of, satisfaction and experiences with spiritual care interventions as well. Firstly, Gomez-Batiste et al. conducted a single-group pretest/posttest design in Spain to assess the effectiveness of interventions within a program entitled “Comprehensive Care for Patients with Advanced Illnesses and their Families”. When comparing patients’ scores on surveys filled out at baseline and after follow-up visits (*n* = 2823), the psychosocial (e.g., anxiety, emotional distress, and mood state) and spiritual dimensions (e.g., meaning in life, peace of mind/forgiveness) were significantly improved. The authors concluded that the interventions by well-trained experts can support the easing of discomfort of patients, particularly those suffering from emotional distress or pain [72].

The previously mentioned Existential Behavioral Therapy [37] was assessed by Stockle et al. in a short, individualized form consisting of two sessions. Based on both quantitative assessment and qualitative interviews with informal caregivers (*n* = 31), the authors concluded that this intervention was feasible and (mostly) acceptable for implementation in practice. The qualitative interviews with selected participants (*n* = 15) showed that they felt stronger in difficult situations and reached an inner state of peace of mind. Additionally, the authors found that focusing on personal sources of strength during the intervention was experienced as helpful for interviewees [65].

Thirdly, based on interviews with bereaved children between eight and 12 years old (*n* = 11), Søfting et al. examined the experiences of death-related rituals by children in Norway. Most of these children were interviewed one year after the death of their family member, and seven were Christian and four were nonbeliever. The researchers found that being included in the rituals was very important for the children, for three main reasons: To be included as a family member, to see for themselves, and to say goodbye to their loved ones [63].

In two other studies, which some may see as psychosocial and others as spiritual care, the benefits of creative therapies were examined. Serra Vila et al. conducted a survey and found that relatives and friends highly valued music therapy, with a mean score of 9.4 (on a scale from 0–10). They reported benefits of this therapy such as perception of support, relaxation, positive mood changes, and facilitation of verbal as well as nonverbal communication [62]. Woolf and Fisher explored hospice patients’ experiences with dance movement psychotherapy in the UK, via four case studies. They found that it could promote patients’ physical, emotional, social, and spiritual wellbeing. Specifically, they found that Dance Movement Psychotherapy (DMP) helped patients to express their loss of sense of self and to reintegrate with their estranged bodies [48].

To summarize, while an RCT on spiritual history taking did not yield any significant results between the intervention and control groups, an RCT on Existential Behavioral Therapy did show a small but significant effect. A short, individualized form of EBT showed feasibility and acceptability for implementation in practice, and in another study the researchers concluded that interventions by well-trained experts can support the easing of discomfort of patients. In addition, death-related rituals appeared to be of great importance for the way in which children cope with bereavement, and creative therapies, such as music therapy and dance movement therapy, seem to provide various benefits, such as social and emotional support and relaxation.

### 3.4. Requirements to Implement Spiritual Care in Palliative Care

This section describes what is required to implement spiritual care in palliative care, which includes the challenges participants in several studies faced.

#### 3.4.1. Spiritual Competency

Caregivers participating in included studies saw spiritual care as part of their role, but this view gave rise to challenges as well [40]. In a Spanish study, for example, 94% of the 191 palliative care professionals saw the provision of spiritual care as part of their role, but only 58% considered themselves competent to provide this type of care [51]. A survey study with 579 Swiss general practitioners found that more than half of the participants saw spiritual competency as important. However, only 38% felt confident in spiritual competency [71]. Dutch palliative consultation teams in a survey (*n* = 25) expressed the desire for training in dealing with spiritual issues [36].

A Norwegian study provided an example of how this might be given shape. A mobile teaching team taught care workers in practice, in order to identify spiritual and existential suffering. They initiated existential and spiritual conversations and conveyed consolation through active silence and being present [45]. For German hospice professionals and volunteers, training in rituals was required to guide hospice patients with a variety of spiritual backgrounds [84]. In another German study, training topics were formulated during a focus group, such as relating meaningfully, referral to chaplains, and voicing and acknowledging your own spirituality [73]. A controlled trial in the Netherlands reported a significant effect of training on health care professionals’ attention to spiritual and existential needs of patients [81].

Several studies pointed at the importance of self-reflection as element of spiritual competency. For example, a survey study with hospice volunteers and their coordinators found that all training programs on spiritual care included self-reflection on personal spirituality as obligatory [54]. In addition, some studies reported differences between hopes, convictions, or needs of patients, their family members and caregivers [38,76], and ICU nurses in a Dutch study on spiritual care stated that they, in order to provide spiritual care, should know themselves and be aware of their own backgrounds, which required reflection and education [58].

#### 3.4.2. Visibility

A Spanish study reported that, in nurses’ images of palliative care, the spiritual dimension was weakly integrated [60], and participants in a mixed-methods study less frequently described the spiritual dimension compared to the physical dimension [70]. Another study reported that participants found spirituality important but rarely discussed the specific tasks of spiritual care [54]. Spiritual caregivers struggled with their visibility as well. For example, spiritual counselors in a Dutch study were motivated to use a structured method of spiritual care because they expected the professionalization and the visibility of their profession to improve [56]. In addition, clergy providing palliative care in the UK had little experience in working with palliative care providers [53], which may reduce their visibility for palliative care providers.

Nevertheless, participants in several studies emphasized the importance of the spiritual caregiver participating in palliative care. Mainly studies conducted in the Netherlands addressed this topic. For example, palliative consultation teams in a survey study stated that the spiritual counselor should be a permanent member of the palliative consultation, and available on call [36]. In another Dutch study, chaplains reported how they supported or could support ICU nurses in the integration of spiritual care in their work [58]. A qualitative study found relevant factors for implementing spiritual care in (palliative) care, which included research based chaplaincy, the context of palliative care in hospitals, and the use of a diagnostic tool. Within the organizational structure it was necessary to have a clear mandate and ownership of spiritual care and good relationships with physicians and managers [83].

A last issue was the visibility of patient groups. A survey among 6263 physicians found that spiritual care was most commonly provided to females, older patients, patients with dementia and patients who died in a nursing home [33]. Another study, examining the experiences of 137 nurses in mental health care, found that only 33% of patients with a life-threatening disease received spiritual care [52]. Furthermore, findings from a qualitative study with sixteen patients with end-stage heart failure living at home in the UK suggest that hospitals did not fully address spiritual concerns of these patients, many of whom struggled with isolation and loneliness [42]. From these three studies, we cannot draw the conclusion that particular groups remained invisible for spiritual care in palliative care, but the included studies do suggest this possibility.

We found that at least two things are required to implement spiritual care in palliative care: spiritual competency and visibility. In (further) developing spiritual competency of health care professionals, education, and self-reflection of caregivers were paramount. Visibility meant that spirituality of patients and caregivers and the experts in spiritual care easily remained invisible in health care organizations, which required spiritual counselors to participate in existing structures, like consultation teams and research projects. It was also found that there may be a risk that some patient groups remain more easily invisible than other groups, which may deprive them of spiritual care.

## 4. Discussion

This study aimed to map the recent empirical literature on spiritual care in palliative care in Europe, and 53 studies were included in the analysis. The results of this analysis suggest that definitions of spirituality often include elements such as meaning and significant relationships. Spiritual care in palliative care means that caregivers pay attention to spirituality, which includes hope. They try to be present, to empower and to bring peace to patients and patients’ relatives. Spiritual care includes creative, narrative, and ritual work. The findings also suggest that spiritual care has positive effects on patients, for example because it eased their discomfort. Nevertheless, the evidence for the effects of spiritual care is low. In order to successfully implement spiritual care in palliative care, in addition, caregivers need to develop their spiritual competency, through education and self-reflection. Additionally, they have to make visible the spiritual dimensions in health care in general, in which spiritual counselors play an important role.

The findings on hope are in line with the thematic review of Sinclair et al. on spirituality within palliative care, in which hope was one of the central topics [26]. In this respect, it is worth mentioning that health care providers should not only look at patients’ hope from the perspective of hope’s truthfulness (realistic perspective). They should also recognize how hope helps their patient because it contributes to their patient’s well-being (functional perspective), or how it fits within the (life) narrative of the patient (narrative perspective) [87]. Our finding of spiritual care as trying to empower patients and their relatives points at the importance of empowerment, which may involve several themes: Self-identity, personalized knowledge in practice and theory, negotiating healthcare and personal relationships, navigation of continued losses, and acknowledgement of terminal illness [88].

Holloway et al. found that there was little data on the promotion of the provision of spiritual care [27], which is confirmed in our study. Our study found several suggestions on what is required to implement spiritual care in palliative care as well. Holloway et al. also highlighted that there was a lack of studies on training, and more specifically, hardly any studies included, in their review, a description of the effects of training on health care providers and patients [27]. This is a topic that deserves further attention in future studies, though our review study included some studies reporting on the (possible) effects of training on caregivers and patients [40,54,73,81,82].

In spite of the growing evidence [4,5], we found only two RCTs conducted in Europe to investigate the effect of spiritual care interventions in palliative care, leaving aside an RCT on training [81,82]. One yielded no demonstrable quantitative effects [67], whereas the other yielded significant but small effects [37]. In this regard, it is worth referring to a recent paper on the state of science of spirituality and palliative care, by researchers working in North America. They stated that the field would benefit from hypothesis-driven outcome research and they pleaded for examining the relationship of such research with key outcomes in palliative care, the use of validated instruments and assessment of potential confounding variables [31]. Based on the findings of our review study, however, we may first need to develop appropriate outcomes since (health-related) quality of life may not fully capture the effects of creative, narrative, or ritual work, which were found to be forms of spiritual care. One possible direction is the development of (instruments to measure) narrative outcomes [89].

Strikingly, French or Italian studies were absent in our review, and except from one study, no studies conducted in Eastern Europe were included. We can only offer tentative explanations for this absence. One explanation is that these studies do exist, but that somehow we did not find them. Another explanation is to look at what these countries have in common, which is is the prominent role of Roman-Catholic Christianity. It may be the case that in these countries spirituality is more easily equated with religion and that the legitimization of spiritual care is based on this tradition. Comparing it to the UK and the Netherlands, where most included studies have been conducted, we recognize that more than one Christian tradition are widely present: Roman-Catholic, Protestant, Anglican and/or Evangelical. More importantly, there is evidence that institutionalized religions in the UK and the Netherlands have transformed into subject based spiritualities [90,91]. In that case, the scope of spiritual care is no longer focused on institutionalized traditions, but on how individuals give meaning to their situation [92].

One of the strengths of this study was its extensive search by making use of many databases, thereby covering references in several languages. While we focused on finding literature from all over Europe, our findings mainly seem to apply to Western Europe, and to a lesser degree to Northern Europe and Spain, which necessitates future studies in other parts of Europe. In addition, we synthesized the findings of European studies and future studies may make cross continent comparisons to identify differences in perspective on spiritual care between Europe and other parts of the world. Another limitation is the synthesis of data that were drawn from studies with various methods, and there are different ideas on how to do such a synthesis [93]. Consequently, it is hard, if not impossible, to assess and compare the quality of studies with so many different designs and theoretical backgrounds. However, synthesizing the results of qualitative studies may strengthen the transferability of the findings [94]. Future studies should, for example, use RCTs to evaluate the effects of spiritual care in palliative care on the receivers of this care. Another limitation or possible bias of our study is that we used a broad definition of spiritual care in order to include as many studies on spiritual care as possible. Reflecting on our strategy, including our search terms, we recognize that there are many similarities with the working definition of spirituality used by the Taskforce Spiritual Care of the EAPC, which is based on a wide consensus in Europe (see Results, spirituality).

We conclude that, based on recent empirical studies, our study has provided insight into spiritual care in palliative care in Europe. A majority of the included studies have been conducted in western European countries, limiting the applicability of our findings to other parts of Europe, like eastern European countries. The findings gained through this study have shown the variety of spiritual care practices and the significance of developing spiritual competencies and visibility of spirituality and spiritual care in healthcare. We conclude that the evidence for spiritual care interventions, based on RCTs, is low. Future studies are necessary to investigate the effects of spiritual care more fully, and to develop outcome measurements that appropriately capture the effects of the variety of spiritual care practices. We hope that our study together with future ones help caregivers to support patients and patients’ relatives to give words to what is meaningful for them in the last phase of their lives.

## Figures and Tables

**Table 1:** Characteristics of the included articles ^a^.

First Author ^b^	Objective	Methods	Participants	*n*	Setting	Country
Brinkman-Stoppelen-burg [33]	To investigate how often PC consultants, pain specialists, psychological experts and spiritual caregivers are involved in caring for patients in the last month of life, and which factors are associated with their involvement	QN: survey	PC team/consultsant (12%), pain specialists (3%), psychologists/psychiatrists (6%), spiritual caregiver (13%), other caregiver (27%)	6263	Various	Netherlands
Burbeck [34]	To assess the involvement of volunteers with direct patient/family contact in UK PC services for children and young people	QN: survey	Hospice providers	21	Hospice	UK
Carrero Planes [35]	To develop a tool to guide the psychosocial and spiritual attention for patients and their families in advanced disease at the end of life, and to analyze the tool’s content from some preliminary results	MM: analysis of responses to the tool	Cancer patients (*n* = 65), main caregivers (*n* = 47)	115	Hospital, home care, day care center	Spain
Ettema [36]	To explore in what way the spiritual dimension of PC is embedded in the palliative consultation teams	QN: survey	Coordinators of palliative consultation teams	25	Palliative consultation teams	Netherlands
Kögler [37]	To investigate the relationship between mindfulness, mental distress, and psychological well-being in informal caregivers, and evaluate if the effects of the intervention were mediated by mindfulness	QN: RCT	Relatives of PC inpatients	130	At home	Germany
Llewellyn [38]	To understand how healthcare professionals conceptualise spirituality among seriously ill children and young people and their families, and their experiences in dealing with spiritual issues that emerge in practice	QL: thematic analysis of workshop	Healthcare professionals working with seriously ill children, among whom nurses (36%), clinical psychologists (16%), chaplains (12%)	25	Various, mainly paediatric community (56%), paediatric hospital (20%)	UK
McTiernan [39]	To explore the lived experience of individuals with terminal cancer receiving PC in Ireland	QL: interviews	Patients with a diagnosis of terminal cancer	8	Hospice or residential settings	Ireland
Paal [40]	To identify spiritual care training courses currently running or planned for the near future	QN: survey	European Association of PC members	36	Various	Various
Papadaniel [41]	To analyse the impacts of changes brought about by an individual illness on the relatives’ employment situation as well as on family dynamics	QL: in-depth interviews	Family members of very ill patients and family members’ colleagues or managers	ca. 80	Various	Switzerland
Ross [42]	To identify the spiritual needs and spiritual support preferences of end-stage heart failure patients/carers and to develop spiritual support guidelines locally	QL: semi structured interviews	Patients with end-stage heart failure	16	At home	UK
Rudilla [43]	To offer evidence on the efficacy of counselling spiritual needs to improve the spirituality of patients attended in several health services	QN: clinical trial	Patients with cancer (87 %) and other patients	131	Home care & hospital	Spain
Thomas [44]	To discover how hospice chaplains understand spirituality in their practice of spiritual care and in their descriptions of their own spirituality	QL: semi structured interviews	Hospice chaplains	25	Hospice	UK
Tornøe [45]	To illuminate a pioneering Norwegian mobile hospice nurse teaching team’s experience with teaching and training care workers in spiritual and existential care for the dying in nursing homes and home care settings	QL: focus group interview	Expert hospice nurses	3	Nursing homes & home care settings	Norway
Tornøe [46]	To describe nurses’ experiences with spiritual and existential care for dying patients in a general hospital	QL: narrative interviews	Nurses, among whom four had degrees in oncology nursing and PC	6	combined medical and oncological ward in general hospital	Norway
Vermandere [47]	To explore nurses’ and physicians’ experiences with the ars moriendi model for spiritual assessment	MM: survey and semi structured interviews	QN: nurses (*n* = 17), family physicians (*n* = 4); QL: nurses (*n* = 19), family physicians (*n* = 5)	24	Palliative home care	Belgium
Woolf [48]	To explore clients’ experiences of dance movement psychotherapy in a day hospice setting	QL: case study	hospice patients	4	Hospice	UK
Bekkema [49]	To explore relevant dimensions of the care relationships in end-of-life care from the perspectives of people with mild intellectual disabilities in the Netherlands	QL: group interviews	persons with an intellectual disability (seven groups)	33	Various	Netherlands
Brinkman-Stoppelen-burg [50]	To study the number of hospitals that have a PC team and the characteristics of these teams	QN: survey	key PC professionals	74	Hospital	Netherlands
Dones Sánchez [51]	To describe and analyze spiritual care conducted by PC teams in our country, from the perspective of committed professionals, and assess potential areas for improvement	QN: survey	PC professionals attending a national conference	191	Various	Spain
Evenblij [52]	To explore nurses’ experiences with and identify barriers to providing PC to psychiatric patients in Dutch mental health facilities	MM: survey, in-depth interviews	Nurses in mental health facilities (QN: *n* = 137; QL: *n* = 9)	137	Mental health facilities	Netherlands
Goodhead [53]	To explore the experiences, attitudes and training in caring for the dying of clergy working in South London, UK	QL: semi structured interviews	Clergy	14	Christian communities	UK
Gratz [54]	To investigate the current practice of spiritual care training in Germany	QN: survey	Hospice volunteers and their coordinators	332	Hospice	Germany
Kienle [55]	To investigate the concepts, therapeutic goals, procedures, and working conditions of integrative oncology doctors in the field of anthroposophic medicine	QL: in-depth interviews	Physicians working in internal medicine (*n* = 17), general practice (*n* = 12), oncology/hematology (*n* = 8), or other settings (*n* = 6) ^c^	35	Hospital or office-based practices	Germany and various
Kruizinga [56]	To understand the lived experience of spiritual counselors working with a new structured method in offering spiritual care to palliative patients in relation to a multidisciplinary health care team	QL: in-depth interviews	Spiritual counselors with various backgrounds: Roman Catholic (*n* = 5), Humanist (*n* = 2), Protestant (*n* = 1), Buddhist (*n* = 1)	9	Hospital	Netherlands
Nolan [57]	To explore the value of chaplaincy work with people who regard themselves as nonreligious	QL: case study	dying man, his family-wife, daughter, sister, and son-in-law-whose religion is secularized	1	Hospice	UK
Noome [58]	To examine the role and responsibilities of intensive care unit (ICU) nurses regarding the spiritual aspects of end-of-life care in the ICU, from the chaplains’ perspectives	QL: focus groups	Hospital chaplains	11	Hospital	Netherlands
Olsman [59]	To describe a relational ethics of hope based on the perspectives of PC patients, their family members and their healthcare professionals	QL: longitudinal semi structured interviews	29 PC patients with incurable cancer (*n* = 11), severe Chronic Obstructive Pulmonary Disease COPD (*n* = 10), severe heart failure (*n* = 8); 19 family members; and 52 healthcare professionals, including physicians (*n* = 24), nurses (*n* = 18), spiritual caregivers (*n* = 10)	100	Various	Netherlands
Ortega Galán [60]	To determine and interpret the nurse perspective on the spiritual dimension of individuals at the end of life	QL: focus groups and semi structured interviews	nurses with responsibility in the areas of health care, management, teaching and research	41	Various	Spain
Rufino Castro [61]	To record spiritual expressions made spontaneously by patients while attending a PC unit	QN: record expressions	Various	276	Various	Spain
Serra Vila [62]	To evaluate the satisfaction of caregivers and the benefits achieved with the intervention with a music therapy (MT) programme implemented in a PC Unit (PCU) in Madrid	MM: surveys	Relatives and friends	100	PC unit	Spain
Søfting [63]	To examine how Norwegian children today are included in death-related rituals after the loss of a parent or sibling, how they experienced their own participation, and to explore the meaning the rituals had for them	QL: semi structured interviews	Children between 8 and 12 years old, most of whom (*n* = 7) were interviewed one year after the death of their family member; they were Christian (*n* = 7) or nonbeliever (*n* = 4)	11	Community	Norway
Steenfeldt [64]	To explore patients’, relatives’, and healthcare professionals’ experience of life and caring practice in two Danish hospice settings	QL: semi structured interviews, observations, field notes	11 patients in a secular or a Christian hospice, 6 relatives, 12 healthcare professionals	29	Hospice	Denmark
Stöckle [65]	To test the feasibility and acceptability of an adapted short-term, individual approach of EBT in preparation for a randomized controlled trial (RCT)	MM: quantitative assessments & qualitative interviews	Informal caregivers of various PC patients	31	Hospital	Germany
van Lancker [66]	To increase the knowledge of the frequency and intensity of symptoms and the treatment interventions in older palliative cancer patients	QN: cross-sectional study (symptom assessments)	Older palliative cancer patients (mean age 75.7), many of whom had a geriatric risk profile	400	Hospital	Belgium
Vermandere [67]	To investigate the effect of a structured spiritual history taking on the spiritual well-being of palliative patients in home care	QN: clustered RCT	204 nurses and 41 physicians	245	Palliative Home Care	Belgium
Willig [68]	To reflect on the use of object elicitation in a phenomenological study of the experience of living with advanced cancer	QL: case study	Patients with advanced cancer	14	Hospital or home	UK
Zenz [69]	To report on PC professionals’ views on advance directives (AD)	QN: survey	276 nurses and 126 physicians, of whom 74.6% had a special qualification in PC	402	Various	Germany
de Graaf [70]	To gain insight into multidimensional care (MC) provided to hospice inpatients by a multiprofessional team (MT) and identify facilitators, to ameliorate multidimensional HC	MM: retrospective (QN) study; focus groups (QL)	36 records of hospice patients in twelve hospices; 4 multiprofessional hospice teams consisting of 2/3 nurses, 1 chaplain and 1 physician	NA	Hospice	Netherlands
Giezendanner [71]	To determine which competencies in end-of-life care are considered important by GPs, to assess GPs’ confidence in these competencies in a European context and their reasons to refer terminally ill patients to a specialist	QN: survey	General practitioners; 80% had been principally responsible for at least one palliative, tumor patient within the last year, 82% for a non-tumor patient; 14% had undergone vocational training in PC	579	Community	Switzerland
Gómez-Batiste [72]	To describe the overall quantitative and qualitative results of a “La Caixa” Foundation and World Health Organization Collaborating Center Program entitled “Comprehensive Care for Patients with Advanced Illnesses and their Families” after four years of experience	MM: survey (both QN and QL)	Professionals (*n* = 133): mainly psychologists and social workers; patients (*n* = 8964); family members (*n* = 11,810).	20907	Various	Spain
Gratz [73]	To define the aims of the course (spiritual care training for hospice volunteers) and its central themes in teaching spirituality to hospice volunteers	QL: focus group	hospice homecare service coordinators/trainers, with teaching experience in spiritual care	8	Hospice	Germany
Kisvetrova [74]	To determine the utilization rate of comfort supporting nursing activities in end-of-life patients in an institutionalized environment in the Czech Republic in relation to the age of the registered nurses (RNs), length of work experience, education level, and type of workplace	QN: survey	Registered nurses; 35.8% worked in long-term care, 29.0% in internal and oncological departments, 26.2% in an ICU, 8.9% in hospice	907	Various	Czech Republic
Loeffen [75]	To develop a functional individualised paediatric PC plan that covers physical, psychological, spiritual and social functioning, with great emphasis on the guideline’s recommendations, advance care planning and patients’ and parents’ preferences and desires	MM: group meetings and survey	patients with brain tumour (*n* = 3), DNA repair-deficiency disorder (*n* = 2), peroxisomal disorder (*n* = 2), congenital heart disease (*n* = 1), or unknown (*n* = 1); 28 individuals: most of whom worked as physician in (a specialized areas of) paediatrics, and two parents	37	Hospitals	Netherlands
Macpherson [76]	To explore difficulties faced by practitioners when assisting a family in the process of preparing a child for the death of a parent	QL: field work	Professionals (mainly medical and nursing staff), patients and families	NA	Various	UK
Olsson [77]	To explore the psychosocial well-being of young people who participated in support groups at a Swedish specialist PC setting	MM: surveys (both QN and QL)	Bereaved young people (aged 16–28 years)	29	PC setting	Sweden
Paal [78]	To analyze the process of spiritual history taking in order to collect expert insights that might be useful for health-care providers interested in integrating the spiritual dimension into their daily work	QL: discussion panel	Spiritual care experts: psychologists, theologians, physicians, nurses, and researchers	11	PC setting	Germany
Shaw [79]	To examine how end-of-life talk is initiated in CALM therapy sessions with advanced cancer patients	QL: conversation analysis	Ten advanced cancer patients, nine social workers, one psychiatrist	20	cancer center	UK
Toivonen [80]	To describe the experiences of nurses supporting spirituality in the care of older people living with dementia	QL: unstructured interviews	Nine RNs and eight assistant nurses, working in home care, outpatient care, or institutional care (public or private)	17	Nursing units for people with dementia	Finland
van de Geer [81]	To measure the effects of a specific spiritual care training on patients’ reports of their perceived care and treatment	QN: controlled trial	PC patients	85	Hospital	Netherlands
van de Geer [82]	To measure effects of a training program on spiritual care in PC based on the guideline of spiritual care in PC	QN: intervention study	Nurses (*n* = 214) and physicians (*n* = 41)	255	Hospital	Netherlands
van de Geer [83]	To explore an implementation strategy for the Dutch multidisciplinary guideline for spiritual care	QL: semi-structured interviews	Chaplains	10?	Hospital	Netherlands
Walker [84]	To explore how spiritual care is provided in hospices and what significance spirituality has in hospices	QL: semi structured interviews	Full-time hospice staff, including nurses (five), the directors of patient care (two), members of the psychiatric service (three), the directors of the hospices (one), chaplains (two) and volunteers (nine)	22	Hospices	Germany
Werner [85]	To explore a real-life medical consultation between a doctor and a patient with incurable cancer, focusing on conveying hope	QL: discourse analysis of one case	Patient with incurable lung cancer and HIV, meeting his specialist in internal medicine	1	University Hospital	Norway

QL, Qualitative study; QN, Quantitative study; MM, Mixed Methods study; NA, not applicable; PC, Palliative Care. ^a^ Several characteristics are quotes drawn from the article’s abstract; ^b^ References are ordered according to their year of publication (references published in 2015 are presented first); and ^c^ some doctors have several specialties and are mentioned twice.

## Supplementary Materials

Supplementary File 1

## Appendix A.

Search Strategy for the different Databases.

## Appendix B.

Process description for Screening Title and abstract.

## Appendix C.

Results of included literature: Definition of spirituality, practice of spiritual care, effectiveness of spiritual care, requirements for implementation of spiritual care.

## References

[1] Saunders C.M., Saunders C.M. (1984). Appropriate treatment, appropriate death. Management of Terminal Malignant Disease.

[2] World Health Organization WHO Definition of Palliative Care. http://www.who.int/cancer/palliative/definition/en.

[3] World Health Organization, Worldwide Palliative Care Alliance Global Atlas of Palliative Care at the End of Life.

[4] MacLean C.D., Susi B., Phifer N., Schultz L., Bynum D., Franco M., Klioze A., Monroe M., Garrett J., Cykert S. (2003). Patient preference for physician discussion and practice of spirituality. J. Gen. Intern. Med..

[5] Best M., Butow P.N., Olver I.N. (2015). Do patients want doctors to talk about spirituality? A systematic literature review. Patient Educ. Couns..

[6] Balboni T.A., Paulk M.E., Balboni M.J., Phelps A.C., Loggers E.T., Wright A.A., Block S.D., Lewis E.F., Peteet J.R., Prigerson H.G. (2010). Provision of spiritual care to patients with advanced cancer: Associations with medical care and Quality of Life near death. J. Clin. Oncol..

[7] Vallurupalli M., Lauderdale K., Balboni M.J., Phelps A.C., Block S.D., Ng A.K., Kachnic L.A., Vanderweele T.J., Balboni T.A. (2012). The role of spirituality and religious coping in the Quality of Life of patients with advanced cancer receiving palliative radiation therapy. J. Support Oncol..

[8] Sinclair S., McConnell S., Raffin Bouchal S., Ager N., Booker R., Enns B., Fung T. (2015). Patient and healthcare perspectives on the importance and efficacy of addressing spiritual issues within an interdisciplinary bone marrow transplant clinic: A qualitative study. BMJ Open.

[9] Kamper R., Van Cleve L., Savedra M. (2010). Children with advanced cancer: Responses to a spiritual Quality of Life interview. J. Spec. Pediatr. Nurs..

[10] Scobie G., Caddell C. (2005). Quality of life at end of life: Spirituality and coping mechanisms in terminally ill patients. Internet J. Pain Symptom Control Palliat. Care.

[11] Zhang B., Nilsson M., Prigerson H. (2012). Factors important to patients’ Quality of Life at the end of life. Arch. Intern. Med..

[12] Bekelman D. (2009). Symptom burden, depression, and spiritual well-being: A comparison of heart failure and advanced cancer patients. J. Gen. Intern Med..

[13] Hills J., Paice J.A., Cameron J.R., Shott S. (2005). Spirituality and distress in palliative care consultation. J. Palliat. Med..

[14] MacKinlay E., Trevitt C. (2012). Finding Meaning in the Experience of Dementia: The Place of Spiritual Reminiscence.

[15] Balboni T., Vanderwerker L.C., Block S.D., Paulk M.E., Lathan C.S., Peteet J.R., Prigerson H.G. (2007). Religiousness and spiritual support among advanced cancer patients and associations with end-of-life treatment preferences and Quality of Life. J. Clin. Oncol..

[16] Balboni T., Balboni M., Paulk M.E., Phelps A., Wright A., Peteet J., Block S., Lathan C., Vanderweele T., Prigerson H. (2011). Support of cancer patients’ spiritual needs and associations with medical care costs at the end of life. Cancer.

[17] Pearce M., Coan A., Herndon J., Koenig H., Abernethy A. (2012). Unmet spiritual care needs impact emotional and spiritual well-being in advanced cancer patients. Support Care Cancer.

[18] Puchalski C., Ferrell B., Virani R., Otis-Green S., Baird P., Bull J.M.A., Chochinov H., George Handzo G., Nelson-Becker H., Prince-Paul M. (2009). Improving the quality of spiritual care as a dimension of palliative care: The report of the Consensus Conference. J. Palliat. Med..

[19] Astrow A.B., Wexler A., Texeira K., He M.K., Sulmasy D.P. (2007). Is failure to meet spiritual needs associated with cancer patients’ perceptions of quality of care and their satisfaction with care?. J. Clin. Oncol..

[20] Balboni M.J., Sullivan A., Amobi A., Phelps A.C., Gorman D.P., Zollfrank A., Peteet J.R., Prigerson H.G., Vanderweele T.J., Balboni T.A. (2013). Why is spiritual care infrequent at the end of life? Spiritual care perceptions among patients, nurses, and physicians and the role of training. J. Clin. Oncol..

[21] Phelps A., Lauderdale K., Alcorn S., Dillinger J., Balboni M.T., Van Wert M., Vanderweele T.J., Balboni T.A. (2012). Addressing spirituality within the care of patients at the end of life: Perspectives of patients with advanced cancer, oncologists, and oncology Nurses. J. Clin. Oncol..

[22] European Association for Palliative Care–Taskforce Spiritual Care in Palliative Care. http://www.eapcnet.eu/Themes/ProjectsTaskforces/EAPCTaskforces/SpiritualCAreinPalliativeCare.aspx.

[23] Spiritual Care Matters: An Introductory Resource for all NHS Scotland Staff. http://www.nes.scot.nhs.uk/education-and-training/by-discipline/spiritual-care/about-spiritual-care/publications/spiritual-care-matters-an-introductory-resource-for-all-nhs-scotland-staff.aspx.

[24] EAPC Blog. https://eapcnet.wordpress.com/2013/11/29/spiritual-care-challenges-in-a-multicultural-and-multireligious-society/.

[25] Global Network for Spirituality and Health. http://smhs.gwu.edu/gwish/global-network.

[26] Sinclair S., Pereira J., Raffin S. (2006). A thematic review of the spirituality literature within palliative care. J. Palliat. Med..

[27] Margaret Holloway Spiritual Care at the End of life: A Systematic Review of the Literature. https://www.gov.uk/government/uploads/system/uploads/attachment_data/file/215798/dh_123804.pdf.

[28] Candy B., Jones L., Varagunam M., Speck P., Tookman A., King M. (2012). Spiritual and religious interventions for well-being of adults in the terminal phase of disease. Cochrane Database Syst. Rev..

[29] Kruizinga R., Hartog I.D., Jacobs M., Daams J.G., Scherer-Rath M., Schilderman J.B., Sprangers M.A., Van Laarhoven H.W. (2016). The effect of spiritual interventions addressing existential themes using a narrative approach on quality of life of cancer patients: A systematic review and meta-analysis. Psychooncology.

[30] Selman L., Young T., Vermandere M., Stirling I., Leget C. (2014). Research subgroup of European Association for Palliative Care Spiritual Care Taskforce. Research priorities in spiritual care: An international survey of palliative care researchers and clinicians. J. Pain. Symptom. Manag..

[31] Steinhauser K., Fitchett G., Handzo G., Johnson K.S., Koenig H., Pargament K., Puchalski C., Sinclair S., Taylor E.J., Balboni T.A. (2017). State of the science of spirituality and palliative care research part I: Definitions and taxonomy, measurement, and outcomes. J. Pain Symptom Manag..

[32] Balboni T.A., Fitchett G., Handzo G., Johnson K.S., Koenig H., Pargament K., Puchalski C., Sinclair S., Taylor E.J., Steinhauser K.E. (2017). State of the science of spirituality and palliative care research part II: Screening, assessment, and interventions. J. Pain Symptom Manag..

[33] Brinkman-Stoppelenburg A., Onwuteaka-Philipsen B.D., van der Heide A. (2015). Involvement of supportive care professionals in patient care in the last month of life. Support Care Cancer.

[34] Burbeck R., Low J., Sampson E.L., Scott R., Bravery R., Candy B. (2015). Volunteer activity in specialist paediatric palliative care: A national survey. BMJ Support Palliat. Care.

[35] Carrero Planes V., Camacho López H., Serrano Font M., Arrué J., Hidalgo F., Hernández M., Sabio P., Navarro Sanz R. (2015). Sentido vital en la enfermedad avanzada: Desarrollo de una herramienta para guiar la atención psicosocial y espiritual en el paciente y familia. Psicooncología.

[36] Ettema E., Wulp M., van Leeuwen R., Leget C. (2015). Embedding of the spiritual dimension in palliative consultation services in the Netherlands: inventory, evaluation, and recommendations. Progr. Palliat. Care.

[37] Kögler M., Brandstätter M., Borasio G.D., Fensterer V., Küchenhoff H., Fegg M.J. (2015). Mindfulness in informal caregivers of palliative patients. Palliat. Support Care.

[38] Llewellyn H., Jones L., Kelly P., Barnes J., O’Gorman B., Craig F., Bluebond-Langner M. (2015). Experiences of healthcare professionals in the community dealing with the spiritual needs of children and young people with life-threatening and life-limiting conditions and their families: Report of a workshop. BMJ Support Palliat. Care.

[39] McTiernan K., O’Connell M. (2015). An interpretative phenomenological analysis exploring the lived experience of individuals dying from terminal cancer in Ireland. Palliat. Support Care.

[40] Paal P., Leget C., Goodhead A. (2015). Spiritual care education: Results from an EAPC survey. Eur. J. Palliat. Care.

[41] Papadaniel Y., Brzak N., Berthod M.A. (2015). Individuals and humanity: Sharing the experience of serious illness. Zeitschrift Fur Ethnologie.

[42] Ross L., Austin J. (2015). Spiritual needs and spiritual support preferences of people with end-stage heart failure and their carers: Implications for nurse managers. J. Nurs. Manag..

[43] Rudilla D., Oliver A., Galiana L., Barreto P. (2015). Espiritualidad en atención paliativa: Evidencias sobre la intervención con counselling. Psychosocial Intervention.

[44] Thomas J. (2015). Hospice chaplains: Talking about spiritual care and avoiding the modern day ‘inquisition’. J. Stud. Spirituality.

[45] Tornøe K., Danbolt L.J., Kvigne K., Sørlie V. (2015). A mobile hospice nurse teaching team’s experience: Training care workers in spiritual and existential care for the dying-A qualitative study. Knowledge, education and training. BMC Palliat. Care.

[46] Tornøe K.A., Danbolt L.J., Kvigne K., Sørlie V. (2015). The challenge of consolation: Nurses’ experiences with spiritual and existential care for the dying-a phenomenological hermeneutical study. BMC Nurs..

[47] Vermandere M., Warmenhoven F., Van Severen E., De Lepeleire J., Aertgeerts B. (2015). The Ars Moriendi Model for spiritual assessment: A mixed-methods evaluation. Oncol. Nurs. Forum.

[48] Woolf S., Fisher P. (2015). The role of dance movement psychotherapy for expression and integration of the self in palliative care. Int. J. Palliat. Nurs..

[49] Bekkema N., de Veer A., Hertogh C.M., Francke A.L. (2016). Perspectives of people with mild intellectual disabilities on care relationships at the end of life: A group interview study. Palliat. Med..

[50] Brinkman-Stoppelenburg A., Boddaert M., Douma J., van der Heide A. (2016). Palliative care in Dutch hospitals: A rapid increase in the number of expert teams, a limited number of referrals. BMC Health Serv. Res..

[51] Dones Sánchez M., Bimbaum N.C., Barbero Gutierrez J., Gomis Bofill C., Balbuena Mora-Figueroa P., Benito Oliver E. (2015). How professionals perceive spiritual care in palliative care teams in Spain?. Medicina Paliativa.

[52] Evenblij K., Widdershoven G.A., Onwuteaka-Philipsen B.D., de Kam H., Pasman H.R. (2016). Palliative care in mental health facilities from the perspective of nurses: A mixed-methods study. J. Psychiatr. Ment. Health Nurs..

[53] Goodhead A., Speck P., Selman L. (2016). ‘I think you just learnt as you went along’-community clergy’s experiences of and attitudes towards caring for dying people: A pilot study. Palliat. Med..

[54] Gratz M., Paal P., Emmelmann M., Roser T. (2016). Spiritual care in the training of hospice volunteers in Germany. Palliat. Support Care.

[55] Kienle G.S., Mussler M., Fuchs D., Kiene H. (2016). Individualized integrative cancer care in anthroposophic medicine: a qualitative study of the concepts and procedures of expert doctors. Integr. Cancer Ther..

[56] Kruizinga R., Helmich E., Schilderman J.B., Scherer-Rath M., van Laarhoven H.W. (2016). Professional identity at stake: A phenomenological analysis of spiritual counselors’ experiences working with a structured model to provide care to palliative cancer patients. Support Care Cancer.

[57] Nolan S. (2016). ‘He needs to talk!’: A chaplain’s case study of nonreligious spiritual care. J. Health Care Chaplain..

[58] Noome M., Beneken Genaamd Kolmer D.M., van Leeuwen E., Dijkstra B.M., Vloet L.C.M. (2017). The role of ICU nurses in the spiritual aspects of end-of-life care in the ICU: An explorative study. Scand. J. Caring Sci..

[59] Olsman E., Willems D., Leget C. (2016). Solicitude: Balancing compassion and empowerment in a relational ethics of hope-an empirical-ethical study in palliative care. Med. Health Care Philos..

[60] Ortega Galán A.M., González De Haro M.D. (2016). The value of the spiritual dimension at the end of life, from nursing professional perspective. Medicina Paliativa.

[61] Rufino Castro M.R., Fernández R.A., Prada Jaimez M.L., Güell Pérez E., Fariñas Balaguer O., Blasco Blasco T., Pascual López A. (2016). Which spiritual sentences are expressed by patients attending a palliative care unit?. Medicina Paliativa.

[62] Serra Vila M., De Luis Molero V.J., Valls i Ballespí J. (2016). Evaluation of a music therapy program in a palliative care unit: The caregivers perspective. Medicina Paliativa.

[63] Søfting G.H., Dyregrov A., Dyregrov K. (2016). Because I’m also part of the family: Children’s participation in rituals after the loss of a parent or sibling. OMEGA.

[64] Steenfeldt V.O. (2016). What is the essence of spiritual care? A Danish hospice perspective. J. Christ. Nurs..

[65] Stöckle H.S., Haarmann-Doetkotte S., Bausewein C., Fegg M.J. (2016). The feasibility and acceptability of short-term, individual existential behavioural therapy for informal caregivers of patients recruited in a specialist palliative care unit. BMC Palliat. Care.

[66] van Lancker A., Beeckman D., van den Noortgate N., Verhaeghe S., van Hecke A. (2017). Frequency and intensity of symptoms and treatment interventions in hospitalized older palliative cancer patients: A multicentre cross-sectional study. J. Adv. Nurs..

[67] Vermandere M., Warmenhoven F., Van Severen E., De Lepeleire J., Aertgeerts B. (2016). Spiritual history taking in palliative home care: A cluster randomized controlled trial. Palliat. Med..

[68] Willig C. (2016). Reflections on the use of object elicitation. Qual. Psychol..

[69] Zenz J., Zenz M. (2017). Survey on German palliative care specialists’ experiences with advance directives. Pain Ther..

[70] de Graaf E., van Klinken M., Zweers D., Teunissen S. (2017). From concept to practice, is multidimensional care the leading principle in hospice care? An exploratory mixed method study. BMJ Support Palliat. Care.

[71] Giezendanner S., Jung C., Banderet H.R., Otte I.C., Gudat H., Haller D.M., Elger B.S., Zemp E., Bally K. (2017). General practitioners’ attitudes towards essential competencies in end of life care: A cross-sectional survey. PLoS ONE.

[72] Gómez-Batiste X., Mateo-Ortega D., Lasmarías C., Novellas A., Espinosa J., Beas E., Ela S., Barbero J. (2017). Enhancing psychosocial and spiritual palliative care: Four-year results of the program of comprehensive care for people with advanced illnesses and their families in Spain. Palliat. Support Care.

[73] Gratz M., Roser T., Paal P. (2017). Hospice volunteers’ spiritual care training: A discussion of core competencies and course aims. Health Soc. Care Chaplain..

[74] Kisvetrová H., Vévodová Š., Školoudík D. (2018). Comfort supporting nursing activities for end of life patients in an institutionalized environment. J. Nurs. Scholarsh..

[75] Loeffen E.A.H., Tissing W.J.E., Schuiling-Otten M.A., de Kruiff C.C., Kremer L.C.M., Verhagen A.A.E. (2018). Pediatric palliative care–individualized care plan working group. Individualised advance care planning in children with life-limiting conditions. Arch. Dis. Child..

[76] Macpherson C. (2017). Difficulties for a practitioner preparing a family for the death of a parent: A narrative inquiry. Mortality.

[77] Olsson M., Lundberg T., Furst C.J., Ohlen J., Forinder U. (2017). Psychosocial well-being of young people who participated in a support group following the loss of a parent to cancer. J. Soc. Work End Life Palliat. Care.

[78] Paal P., Frick E., Roser T., Jobin G. (2017). Expert Discussion on taking a spiritual history. J. Palliat. Care.

[79] Shaw C., Chrysikou V., Davis S., Gessler S., Rodin G., Lanceley A. (2017). Inviting end of life talk in initial CALM therapy sessions: A conversation analytic study. Patient Educ. Couns..

[80] Toivonen K., Charalambous A., Suhonen R. (2017). Supporting spirituality in the care of older people living with dementia: A hermeneutic phenomenological inquiry into nurses’ experiences. Scand. J. Caring Sci..

[81] van de Geer J., Groot M., Andela R., Leget C., Prins J., Vissers K., Zock H. (2017). Training hospital staff on spiritual care in palliative care influences patient-reported outcomes: Results of a quasi-experimental study. Palliat. Med..

[82] van de Geer J., Veeger N., Groot M., Zock H., Leget C., Prins J., Vissers K. (2018). Multidisciplinary training on spiritual care for patients in palliative care trajectories improves the attitudes and competencies of hospital medical staff: Results of a quasi-experimental study. Am. J. Hosp. Palliat. Care.

[83] van de Geer J., Visser A., Zock H., Leget C., Prins J., Vissers K. (2018). Improving spiritual care in hospitals in the Netherlands: What do health care chaplains involved in an action-research study report?. J. Health Care Chaplain..

[84] Walker A., Breitsameter C. (2017). The provision of spiritual care in hospices: A study in four hospices in North Rhine-Westphalia. J. Relig. Health.

[85] Werner A., Steihaug S. (2017). Conveying hope in consultations with patients with life-threatening diseases: The balance between supporting and challenging the patient. Scand. J. Prim. Health Care.

[86] Nolan S., Saltmarsh P., Leget C. (2011). Spiritual care in palliative care: Working towards an EAPC Task Force. Eur. J. Palliat. Care.

[87] Olsman E., Leget C., Onwuteaka-Philipsen B., Willems D. (2014). Should palliative care patients’ hope be truthful, helpful or valuable? An interpretative synthesis of literature describing healthcare professionals’ perspectives on hope of palliative care patients. Palliat. Med..

[88] Wakefield D., Bayly J., Selman L.E., Firth A.M., Higginson I.J., Murtagh F.E.M. (2018). Patient empowerment, what does it mean for adults in the advanced stages of a life-limiting illness: A systematic review using critical interpretive synthesis. Palliat. Med..

[89] Tromp T., Ganzevoort R.R., Francis L., Astley J., Robbins M. (2009). Narrative competence and the meaning of life: Measuring the quality of life stories in a project on care for the elderly. Empirical Theology in Texts and Tables.

[90] Heelas P., Woodhead L. (2005). The Spiritual Revolution: Why Religion Is Giving Way to Spirituality.

[91] Sengers E. (2005). The Dutch and their gods. Secularization and Transformation of Religion in the Netherlands since 1950.

[92] Olsman E., Willems D. (2017). From religious to existential issues: The implications for GPs. Eur. J. Gen. Pract..

[93] Dixon-Woods M., Agarwal S., Jones D., Young B., Sutton A. (2005). Synthesising qualitative and quantitative evidence: A review of possible methods. J. Health Serv. Res. Policy.

[94] Finfgeld-Connett D. (2010). Generalizability and transferability of meta-synthesis research findens. J. Adv. Nurs..

