# Supplementary material for: Spiritual Care in Palliative Care: A Systematic Review of the Recent European Literature
Source: Med Sci (Basel). 2019 Feb 7;7(2):25. doi: 10.3390/medsci7020025 (PMC6409788; doi:10.3390/medsci7020025)
Supplement: Supplementary File 1 [file medsci-07-00025-s001.pdf]

Appendix 1. Search Strategy Databases

PubMed Strategy

Search	Add to builder	Query
#6	Add	Search **#5 AND ("2010"[Date - Publication] : "2017"[Date - Publication])**
#5	Add	Search **#1 AND #4**
#4	Add	Search **#2 OR #3**
#3	Add	Search **1049-9091[IS] OR 1472-684x[IS] OR 1357-6321[IS] OR 1536-0539[IS] OR 0825-8597[IS] OR 1557-7740[IS] OR 1552-4264[IS] OR 1478-9523[IS] OR 1477-030X[IS] OR 0749-1565[IS] OR 0742-969X[IS] OR 1544-6794[IS] OR 0941-4355[IS] OR 1873-6513[IS] OR 0145-7624[IS] OR 1091-7683[IS] OR 0030-2228[IS]**
#2	Add	Search **"Palliative Care"[Mesh] OR "Terminal Care"[Mesh] OR "Hospice Care"[Mesh] OR "Attitude to Death"[Mesh] OR "Aged, 80 and over"[Mesh] OR "Aged"[Mesh] OR "Chronic Disease"[Mesh] OR "Hospitals, Chronic Disease"[Mesh] OR palliati*[tiab] OR terminal[tiab] OR "end of life"[tiab] OR "limited life"[tiab] OR hospice*[tiab] OR dying*[tiab]**
#1	Add	Search **"Spirituality"[Mesh:noexp] OR "Spiritual Therapies"[Mesh:noexp] OR spiritual*[tiab] OR religi*[tiab] OR (meaning[tiab] AND (life[tiab] OR death[tiab])) OR pastoral[tiab] OR faith[tiab]**

ATLA Search Strategy

#	Query
S8	S7<br><br>Limiters - Year Published: 2015-2017
S7	S3 AND S6
S6	S4 OR S5
S5	TI (palliative OR terminal OR "end of life" OR "limited life" OR palliati* OR hospice* OR dying*) OR AB (palliative OR terminal OR "end of life" OR "limited life" OR palliati* OR hospice* OR dying*)
S4	DE "Palliative treatment" OR DE "Terminal care" OR DE "Aged" OR DE "Frail aged"
S3	S1 OR S2
S2	TI (spiritual* OR religi* OR (meaning AND (life OR death)) OR pastoral OR faith)) OR AB (spiritual* OR religi* OR (meaning AND (life OR death)) OR pastoral OR faith)

S1	DE "Spirituality"

CINAHL Search Strategy

#	Query
S8	S7 Limiters - Published Date: 20150101-20171231
S7	S3 AND S6
S6	S4 OR S5
S5	TI (palliative OR terminal OR "end of life" OR "limited life" OR palliati* OR hospice* OR dying*) OR AB (palliative OR terminal OR "end of life" OR "limited life" OR palliati* OR hospice* OR dying*)
S4	MH "Palliative Care" OR MH "Terminal Care" OR MH "Hospice Care" OR MH "Hospice and Palliative Nursing" OR MH "Terminally Ill Patients" OR MH "Attitude to Death" OR MH "Chronic Disease" OR MH "Aged+"
S3	S1 OR S2
S2	TI (spiritual* OR religi* OR (meaning AND (life OR death)) OR pastoral OR faith)) OR AB (spiritual* OR religi* OR (meaning AND (life OR death)) OR pastoral OR faith)
S1	MH "Spirituality" OR MH "Spiritual Care" OR MH "Religion and Religions+"

PsycINFO Search Strategy

#	Query
S8	S7<br><br>Limiters - Publication Year: 2015-2017
S7	S3 AND S6

S6	S4 OR S5
S5	TI (palliative OR terminal OR "end of life" OR "limited life" OR palliati* OR hospice* OR dying*) OR AB (palliative OR terminal OR "end of life" OR "limited life" OR palliati* OR hospice* OR dying*)
S4	DE "Palliative Care" OR DE "Long Term Care" OR DE "Chronic Illness"
S3	S1 OR S2
S2	TI (spiritual* OR religi* OR (meaning AND (life OR death)) OR pastoral OR faith)) OR AB (spiritual* OR religi* OR (meaning AND (life OR death)) OR pastoral OR faith)
S1	DE "Spirituality"

ERIC Search Strategy

#	Query
S8	S7<br><br>Limiters - Date Published: 20100101-20171231
S7	S3 AND S6
S6	S4 OR S5
S5	TI (palliative OR terminal OR "end of life" OR "limited life" OR palliati* OR hospice* OR dying*) OR AB (palliative OR terminal OR "end of life" OR "limited life" OR palliati* OR hospice* OR dying*)
S4	((DE "Hospices (Terminal Care)") OR (DE "Chronic Illness") OR DE "Older Adults"
S3	S1 OR S2
S2	TI (spiritual* OR religi* OR (meaning AND (life OR death)) OR pastoral OR faith)) OR AB (spiritual* OR religi* OR (meaning AND (life OR death)) OR pastoral OR faith)

S1	DE "Religious Factors"

Web of Science Search Strategy:

Set	Save History / Create Alert · Open Saved History	Edit Sets	Combine Sets AND OR	Delete Sets
# 4	#1 AND #2 *Indexes=SCI-EXPANDED, SSCI, A&HCI, ESCI Timespan=2015-2017*	Edit	Select to combine sets.	Select to delete this set.
# 3	#1 AND #2 *Indexes=SCI-EXPANDED, SSCI, A&HCI, ESCI Timespan=All years*	Edit	Select to combine sets.	Select to delete this set.
# 2	TS=((palliative OR terminal OR "end of life" OR "limited life" OR palliati* OR hospice* OR dying*)) *Indexes=SCI-EXPANDED, SSCI, A&HCI, ESCI Timespan=All years*	Edit	Select to combine sets.	Select to delete this set.
# 1	TS=((spiritual* OR religi* OR (meaning AND (life OR death)) OR pastoral OR faith)) *Indexes=SCI-EXPANDED, SSCI, A&HCI, ESCI Timespan=All years*	Edit	Select to combine sets.	Select to delete this set.
			AND OR	

Embase Session Strategy

No.	Query
#4	**#1** AND **#2** AND [2010-2017]/py
#3	**#1** AND **#2**
#2	**'palliative nursing'**/exp OR **'terminal care'**/de OR **'hospice care'**/exp OR **'hospice nursing'**/exp OR **'terminally ill patient'**/exp OR **'dying'**/exp OR **'attitude to death'**/exp

No.	Query
	OR **'aged'**/exp OR **'chronic disease'**/exp OR **terminal**:ab,ti OR **'end of life'**:ab,ti OR **'limited life'**:ab,ti OR **palliati***:ab,ti OR **hospice***:ab,ti OR **dying***:ab,ti
#1	**'religion'**/exp OR **spiritual***:ab,ti OR **religi***:ab,ti OR (**meaning**:ab,ti AND (**life**:ab,ti OR **death**:ab,ti)) OR **pastoral**:ab,ti OR **faith**:ab,ti

IBSS Search Strategy

S4

(SU.EXACT("Spirituality") OR ti((spiritual* OR religi* OR (meaning AND (life OR death)) OR pastoral OR faith)) OR ab((spiritual* OR religi* OR (meaning AND (life OR death)) OR pastoral OR faith))) AND (SU.EXACT("Palliative care") OR (SU.EXACT("Aged") OR SU.EXACT("Care of the aged")) OR ti((palliative OR terminal OR "end of life" OR "limited life" OR palliati* OR hospice* OR dying*)) OR ab((palliative OR terminal OR "end of life" OR "limited life" OR palliati* OR hospice* OR dying*)))

Limits publication date 2015-2017

S3

(SU.EXACT("Spirituality") OR ti((spiritual* OR religi* OR (meaning AND (life OR death)) OR pastoral OR faith)) OR ab((spiritual* OR religi* OR (meaning AND (life OR death)) OR pastoral OR faith))) AND (SU.EXACT("Palliative care") OR (SU.EXACT("Aged") OR SU.EXACT("Care of the aged")) OR ti((palliative OR terminal OR "end of life" OR "limited life" OR palliati* OR hospice* OR dying*)) OR ab((palliative OR terminal OR "end of life" OR "limited life" OR palliati* OR hospice* OR dying*)))

S2

SU.EXACT("Palliative care") OR (SU.EXACT("Aged") OR SU.EXACT("Care of the aged")) OR ti((palliative OR terminal OR "end of life" OR "limited life" OR palliati* OR hospice* OR dying*)) OR ab((palliative OR terminal OR "end of life" OR "limited life" OR palliati* OR hospice* OR dying*))

S1

SU.EXACT("Spirituality") OR ti((spiritual* OR religi* OR (meaning AND (life OR death)) OR pastoral OR faith)) OR ab((spiritual* OR religi* OR (meaning AND (life OR death)) OR pastoral OR faith))

PICARTA Search Strategy

set	actie

9 zoeken [en]s4 en s7

verkleinen\9001 AD (articles)

8 zoeken [en]s4 en s7

7 zoeken [en]s5 of s6

6 zoeken [en](alle woorden)terminaalfilter instellingen

vergroten(alle woorden)levenseindefilter instellingen

vergroten(alle woorden)hospice*filter instellingen

vergroten(alle woorden)sterven*filter instellingen

5 zoeken [en](alle woorden)palliati*filter instellingen

4 zoeken [en]s1 of s2 of s3

3 zoeken [en](alle woorden)spiritu*filter instellingen

vergroten(alle woorden)religi*filter instellingen

vergroten(alle woorden)pastora*filter instellingen

vergroten(alle woorden)gelooffilter instellingen

2 zoeken [en](alle woorden)zingevingfilter instellingen

verkleinen(alle woorden)dood*filter instellingen

1 zoeken [en](alle woorden)zingevingfilter instellingen

verkleinen(alle woorden)leven*filter instellingen

SCIELO Search Strategy

Set	Save History / Create Alert Open Saved History	Combine Sets<br>AND OR	Delete Sets
# 4	#1 AND #2<br>*Indexes=SCIELO Timespan=2015-2017*	Select to combine sets.	Select to delete this set.
# 3	#2 AND #1<br>*Indexes=SCIELO Timespan=All years*	Select to combine sets.	Select to delete this set.

# 2	**TOPIC:** ((Paliati* OR termina* OR ((final OR fim) AND vida) OR "vida limitada" OR hospic* OR moribund* OR morrend*))<br><br>*Indexes=SCIELO Timespan=All years*	Select to combine sets.	Select to delete this set.
# 1	**TOPIC:** ((espiritual* OR religi* OR ((significado OR sentido) AND (vida OR muerte OR morte)) OR pastoral OR fe))<br><br>*Indexes=SCIELO Timespan=All years*	Select to combine sets.	Select to delete this set.

LILACS + IBECS Search strategy

http://search.bvsalud.org/portal/advanced/?lang=en

In LILACS

"spirituality" OR "spiritualism" OR "pastoral care" (in Subject descriptor)

"palliative care" OR "palliative care nursing" OR "hospice and palliative care nursing" OR "palliative treatment" OR "terminal care" OR "hospice care" OR "hospices" (in Subject descriptor)

spiritual$ OR espiritual$ OR religi$ OR (significado OR sentido) AND (vida OR muerte OR morte) OR pastoral OR fe (in title, abstract,subject)

AND

Palliati$ OR Paliati$ OR termina$ OR **((**fim OR final) AND vida) OR (vida AND limitada) OR hospic$ OR morrend$ OR moribund$ (in title, abstract,subject)

13175
Limitation LILACS + IBECS
Limitation 2015-2017

Scopus search strategy

4 (TITLE-ABS-KEY (((spiritual* OR religi* OR (meaning AND (life OR death)) OR pastoral OR faith)))) AND (TITLE-ABS-KEY (((palliative OR terminal OR "end of life" OR "limited life" OR palliati* OR hospice* OR dying*)))) AND (LIMIT-TO (PUBYEAR , 2017) OR LIMIT-TO (PUBYEAR , 2016) OR LIMIT-TO (PUBYEAR , 2015)

3 (TITLE-ABS-KEY (((spiritual* OR religi* OR (meaning AND (life OR death)) OR pastoral OR faith)))) AND (TITLE-ABS-KEY (((palliative OR terminal OR "end of life" OR "limited life" OR palliati* OR hospice* OR dying*))))

2 TITLE-ABS-KEY (((palliative OR terminal OR "end of life" OR "limited life" OR palliati* OR hospice* OR dying*)))

1 TITLE-ABS-KEY (((spiritual* OR religi* OR (meaning AND (life OR death)) OR pastoral OR faith)))

PubPsych (Psyndex) search strategy

https://pubpsych.zpid.de/pubpsych/

https://www.pubpsych.de/

((Spiritual* OR Spirituel* OR Religi* OR Seelsorge* OR FT=Glaube) OR ((Leben* OR Tod OR Sterben) AND (Sinn OR Bedeutung))) AND ((Palliati* OR Sterbe* OR Lebensende* OR Hospiz* OR (Begrenzt* AND Leben*)))

Ethmed at IDEM searc strategy

http://www.idem.uni-goettingen.de/en/ethmed.html

((Spiritual* OR Spirituel* OR Religi* OR Seelsorge* OR FT=Glaube) OR ((Leben* OR Tod OR Sterben) AND (Sinn OR Bedeutung))) AND ((Palliati* OR Sterbe* OR Lebensende* OR Hospiz* OR (Begrenzt* AND Leben*)))

Appendix 1. Search Strategy Databases

PubMed Strategy

Search	Add to builder	Query
#6	Add	Search **#5 AND ("2010"[Date - Publication] : "2017"[Date - Publication])**
#5	Add	Search **#1 AND #4**
#4	Add	Search **#2 OR #3**
#3	Add	Search **1049-9091[IS] OR 1472-684x[IS] OR 1357-6321[IS] OR 1536-0539[IS] OR 0825-8597[IS] OR 1557-7740[IS] OR 1552-4264[IS] OR 1478-9523[IS] OR 1477-030X[IS] OR 0749-1565[IS] OR 0742-969X[IS] OR 1544-6794[IS] OR 0941-4355[IS] OR 1873-6513[IS] OR 0145-7624[IS] OR 1091-7683[IS] OR 0030-2228[IS]**
#2	Add	Search **"Palliative Care"[Mesh] OR "Terminal Care"[Mesh] OR "Hospice Care"[Mesh] OR "Attitude to Death"[Mesh] OR "Aged, 80 and over"[Mesh] OR "Aged"[Mesh] OR "Chronic Disease"[Mesh] OR "Hospitals, Chronic Disease"[Mesh] OR palliati*[tiab] OR terminal[tiab] OR "end of life"[tiab] OR "limited life"[tiab] OR hospice*[tiab] OR dying*[tiab]**
#1	Add	Search **"Spirituality"[Mesh:noexp] OR "Spiritual Therapies"[Mesh:noexp] OR spiritual*[tiab] OR religi*[tiab] OR (meaning[tiab] AND (life[tiab] OR death[tiab])) OR pastoral[tiab] OR faith[tiab]**

ATLA Search Strategy

#	Query
S8	S7<br><br>Limiters - Year Published: 2015-2017
S7	S3 AND S6
S6	S4 OR S5
S5	TI (palliative OR terminal OR "end of life" OR "limited life" OR palliati* OR hospice* OR dying*) OR AB (palliative OR terminal OR "end of life" OR "limited life" OR palliati* OR hospice* OR dying*)
S4	DE "Palliative treatment" OR DE "Terminal care" OR DE "Aged" OR DE "Frail aged"
S3	S1 OR S2
S2	TI (spiritual* OR religi* OR (meaning AND (life OR death)) OR pastoral OR faith)) OR AB (spiritual* OR religi* OR (meaning AND (life OR death)) OR pastoral OR faith)

S1	DE "Spirituality"

CINAHL Search Strategy

#	Query
S8	S7 Limiters - Published Date: 20150101-20171231
S7	S3 AND S6
S6	S4 OR S5
S5	TI (palliative OR terminal OR "end of life" OR "limited life" OR palliati* OR hospice* OR dying*) OR AB (palliative OR terminal OR "end of life" OR "limited life" OR palliati* OR hospice* OR dying*)
S4	MH "Palliative Care" OR MH "Terminal Care" OR MH "Hospice Care" OR MH "Hospice and Palliative Nursing" OR MH "Terminally Ill Patients" OR MH "Attitude to Death" OR MH "Chronic Disease" OR MH "Aged+"
S3	S1 OR S2
S2	TI (spiritual* OR religi* OR (meaning AND (life OR death)) OR pastoral OR faith)) OR AB (spiritual* OR religi* OR (meaning AND (life OR death)) OR pastoral OR faith)
S1	MH "Spirituality" OR MH "Spiritual Care" OR MH "Religion and Religions+"

PsycINFO Search Strategy

#	Query
S8	S7<br><br>Limiters - Publication Year: 2015-2017
S7	S3 AND S6

S6	S4 OR S5
S5	TI (palliative OR terminal OR "end of life" OR "limited life" OR palliati* OR hospice* OR dying*) OR AB (palliative OR terminal OR "end of life" OR "limited life" OR palliati* OR hospice* OR dying*)
S4	DE "Palliative Care" OR DE "Long Term Care" OR DE "Chronic Illness"
S3	S1 OR S2
S2	TI (spiritual* OR religi* OR (meaning AND (life OR death)) OR pastoral OR faith)) OR AB (spiritual* OR religi* OR (meaning AND (life OR death)) OR pastoral OR faith)
S1	DE "Spirituality"

ERIC Search Strategy

#	Query
S8	S7<br><br>Limiters - Date Published: 20100101-20171231
S7	S3 AND S6
S6	S4 OR S5
S5	TI (palliative OR terminal OR "end of life" OR "limited life" OR palliati* OR hospice* OR dying*) OR AB (palliative OR terminal OR "end of life" OR "limited life" OR palliati* OR hospice* OR dying*)
S4	((DE "Hospices (Terminal Care)") OR (DE "Chronic Illness") OR DE "Older Adults"
S3	S1 OR S2
S2	TI (spiritual* OR religi* OR (meaning AND (life OR death)) OR pastoral OR faith)) OR AB (spiritual* OR religi* OR (meaning AND (life OR death)) OR pastoral OR faith)

S1	DE "Religious Factors"

Web of Science Search Strategy:

Set	Save History / Create Alert · Open Saved History	Edit Sets	Combine Sets<br>AND OR	Delete Sets
# 4	#1 AND #2<br>*Indexes=SCI-EXPANDED, SSCI, A&HCI, ESCI Timespan=2015-2017*	Edit	Select to combine sets.	Select to delete this set.
# 3	#1 AND #2<br>*Indexes=SCI-EXPANDED, SSCI, A&HCI, ESCI Timespan=All years*	Edit	Select to combine sets.	Select to delete this set.
# 2	TS=((palliative OR terminal OR "end of life" OR "limited life" OR palliati* OR hospice* OR dying*))<br>*Indexes=SCI-EXPANDED, SSCI, A&HCI, ESCI Timespan=All years*	Edit	Select to combine sets.	Select to delete this set.
# 1	TS=((spiritual* OR religi* OR (meaning AND (life OR death)) OR pastoral OR faith))<br>*Indexes=SCI-EXPANDED, SSCI, A&HCI, ESCI Timespan=All years*	Edit	Select to combine sets.	Select to delete this set.
			AND OR	

Embase Session Strategy

No.	Query
#4	**#1** AND **#2** AND [2010-2017]/py
#3	**#1** AND **#2**
#2	**'palliative nursing'**/exp OR **'terminal care'**/de OR **'hospice care'**/exp OR **'hospice nursing'**/exp OR **'terminally ill patient'**/exp OR **'dying'**/exp OR **'attitude to death'**/exp

No.	Query
	OR **'aged'**/exp OR **'chronic disease'**/exp OR **terminal**:ab,ti OR **'end of life'**:ab,ti OR **'limited life'**:ab,ti OR **palliati***:ab,ti OR **hospice***:ab,ti OR **dying***:ab,ti
#1	**'religion'**/exp OR **spiritual***:ab,ti OR **religi***:ab,ti OR (**meaning**:ab,ti AND (**life**:ab,ti OR **death**:ab,ti)) OR **pastoral**:ab,ti OR **faith**:ab,ti

IBSS Search Strategy

S4

(SU.EXACT("Spirituality") OR ti((spiritual* OR religi* OR (meaning AND (life OR death)) OR pastoral OR faith)) OR ab((spiritual* OR religi* OR (meaning AND (life OR death)) OR pastoral OR faith))) AND (SU.EXACT("Palliative care") OR (SU.EXACT("Aged") OR SU.EXACT("Care of the aged")) OR ti((palliative OR terminal OR "end of life" OR "limited life" OR palliati* OR hospice* OR dying*)) OR ab((palliative OR terminal OR "end of life" OR "limited life" OR palliati* OR hospice* OR dying*)))

Limits publication date 2015-2017

S3

(SU.EXACT("Spirituality") OR ti((spiritual* OR religi* OR (meaning AND (life OR death)) OR pastoral OR faith)) OR ab((spiritual* OR religi* OR (meaning AND (life OR death)) OR pastoral OR faith))) AND (SU.EXACT("Palliative care") OR (SU.EXACT("Aged") OR SU.EXACT("Care of the aged")) OR ti((palliative OR terminal OR "end of life" OR "limited life" OR palliati* OR hospice* OR dying*)) OR ab((palliative OR terminal OR "end of life" OR "limited life" OR palliati* OR hospice* OR dying*)))

S2

SU.EXACT("Palliative care") OR (SU.EXACT("Aged") OR SU.EXACT("Care of the aged")) OR ti((palliative OR terminal OR "end of life" OR "limited life" OR palliati* OR hospice* OR dying*)) OR ab((palliative OR terminal OR "end of life" OR "limited life" OR palliati* OR hospice* OR dying*))

S1

SU.EXACT("Spirituality") OR ti((spiritual* OR religi* OR (meaning AND (life OR death)) OR pastoral OR faith)) OR ab((spiritual* OR religi* OR (meaning AND (life OR death)) OR pastoral OR faith))

PICARTA Search Strategy

set	actie

9 zoeken [en]s4 en s7

verkleinen\9001 AD (articles)

8 zoeken [en]s4 en s7

7 zoeken [en]s5 of s6

6 zoeken [en](alle woorden)terminaalfilter instellingen

vergroten(alle woorden)levenseindefilter instellingen

vergroten(alle woorden)hospice*filter instellingen

vergroten(alle woorden)sterven*filter instellingen

5 zoeken [en](alle woorden)palliati*filter instellingen

4 zoeken [en]s1 of s2 of s3

3 zoeken [en](alle woorden)spiritu*filter instellingen

vergroten(alle woorden)religi*filter instellingen

vergroten(alle woorden)pastora*filter instellingen

vergroten(alle woorden)gelooffilter instellingen

2 zoeken [en](alle woorden)zingevingfilter instellingen

verkleinen(alle woorden)dood*filter instellingen

1 zoeken [en](alle woorden)zingevingfilter instellingen

verkleinen(alle woorden)leven*filter instellingen

SCIELO Search Strategy

Set	Save History / Create Alert Open Saved History	Combine Sets AND OR	Delete Sets
# 4	#1 AND #2 *Indexes=SCIELO Timespan=2015-2017*	Select to combine sets.	Select to delete this set.
# 3	#2 AND #1 *Indexes=SCIELO Timespan=All years*	Select to combine sets.	Select to delete this set.

# 2	**TOPIC:** ((Paliati* OR termina* OR ((final OR fim) AND vida) OR "vida limitada" OR hospic* OR moribund* OR morrend*))<br><br>*Indexes=SCIELO Timespan=All years*	Select to combine sets.	Select to delete this set.
# 1	**TOPIC:** ((espiritual* OR religi* OR ((significado OR sentido) AND (vida OR muerte OR morte)) OR pastoral OR fe))<br><br>*Indexes=SCIELO Timespan=All years*	Select to combine sets.	Select to delete this set.

LILACS + IBECS Search strategy

http://search.bvsalud.org/portal/advanced/?lang=en

In LILACS

"spirituality" OR "spiritualism" OR "pastoral care" (in Subject descriptor)

"palliative care" OR "palliative care nursing" OR "hospice and palliative care nursing" OR "palliative treatment" OR "terminal care" OR "hospice care" OR "hospices" (in Subject descriptor)

spiritual$ OR espiritual$ OR religi$ OR (significado OR sentido) AND (vida OR muerte OR morte) OR pastoral OR fe (in title, abstract,subject)

AND

Palliati$ OR Paliati$ OR termina$ OR **((**fim OR final) AND vida) OR (vida AND limitada) OR hospic$ OR morrend$ OR moribund$ (in title, abstract,subject)

13175
Limitation LILACS + IBECS
Limitation 2015-2017

Scopus search strategy

4 (TITLE-ABS-KEY (((spiritual* OR religi* OR (meaning AND (life OR death)) OR pastoral OR faith)))) AND (TITLE-ABS-KEY (((palliative OR terminal OR "end of life" OR "limited life" OR palliati* OR hospice* OR dying*)))) AND (LIMIT-TO (PUBYEAR , 2017) OR LIMIT-TO (PUBYEAR , 2016) OR LIMIT-TO (PUBYEAR , 2015)

3 (TITLE-ABS-KEY (((spiritual* OR religi* OR (meaning AND (life OR death)) OR pastoral OR faith)))) AND (TITLE-ABS-KEY (((palliative OR terminal OR "end of life" OR "limited life" OR palliati* OR hospice* OR dying*))))

2 TITLE-ABS-KEY (((palliative OR terminal OR "end of life" OR "limited life" OR palliati* OR hospice* OR dying*)))

1 TITLE-ABS-KEY (((spiritual* OR religi* OR (meaning AND (life OR death)) OR pastoral OR faith)))

PubPsych (Psyndex) search strategy

https://pubpsych.zpid.de/pubpsych/

https://www.pubpsych.de/

((Spiritual* OR Spirituel* OR Religi* OR Seelsorge* OR FT=Glaube) OR ((Leben* OR Tod OR Sterben) AND (Sinn OR Bedeutung))) AND ((Palliati* OR Sterbe* OR Lebensende* OR Hospiz* OR (Begrenzt* AND Leben*)))

Ethmed at IDEM searc strategy

http://www.idem.uni-goettingen.de/en/ethmed.html

((Spiritual* OR Spirituel* OR Religi* OR Seelsorge* OR FT=Glaube) OR ((Leben* OR Tod OR Sterben) AND (Sinn OR Bedeutung))) AND ((Palliati* OR Sterbe* OR Lebensende* OR Hospiz* OR (Begrenzt* AND Leben*)))

Appendix 2. Screening Title and Abstract

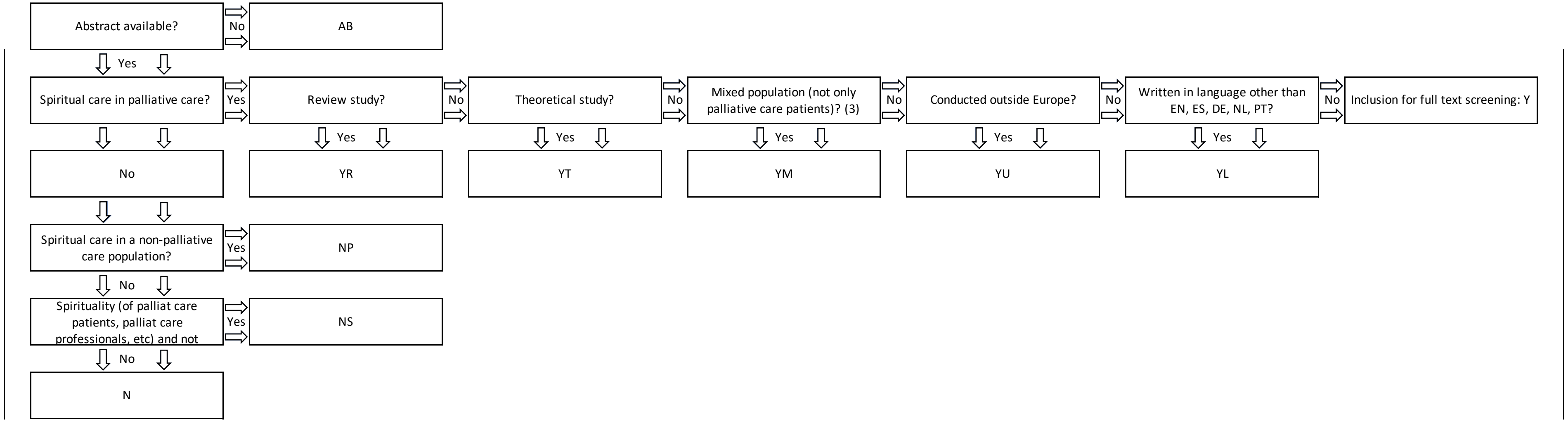

Appendix 3. Definition of spirituality, practice of spiritual care, effectiveness of spiritual care, requirements for implementation of spiritual care

1st author & year	Results on spiritual care	Definition Spirituality (in whole article)	Practice of Spiritual Care in Palliative Care (in results section)	Effectiveness of spiritual care (in results section)	Requirements for implementation of spiritual care (in results section)
Brinkman-Stoppelenburg 2015	A palliative care team or consultant had been involved in the last month of life in 12 % of all patients for whom death was expected; this percentage was 3 % for pain specialists, 6% for psychologists or psychiatrists and 13% for spiritual caregivers. Involvement of palliative care or pain specialists was most common in younger patients, in patients with cancer and in patients who died at home. Involvement of psychological or spiritual caregivers was most common in older patients, in females, in patients with dementia and in patients who died in a nursing home. Involvement of supportive caregivers was also associated with the use of morphine and end-of-life decisions. Conclusion: Supportive care professionals are involved in end-of-life care in about a quarter of all non-suddenly dying patients. Their involvement is related to the setting where patients die, to the patient's characteristics and to complex ethical decision-making.	None	Involvement of psychological or spiritual caregivers was most common in older patients, in females, in patients with dementia and in patients who died in a nursing home.		
Burbeck 2015	A total of 21 providers covering 31 hospices/palliative care services responded (30 evaluable responses). Referral age limit was 16–19 years in 23 services and 23–35 years in seven services; three services were Hospice at Home or home care only. Per service, there was a median of 25 volunteers with direct patient/ family contact. Services providing only home care involved fewer volunteers than hospices with beds. Volunteers entirely ran some services, notably complementary therapy and pastoral/faith-based care. Complementary therapists, school teachers and spiritual care workers most commonly volunteered their professional skills. Volunteers undertook a wide range of activities including emotional support and recreational activities with children and siblings. Conclusions: This is the most detailed national survey of volunteer activity in palliative care services for children and young people to date. It highlights the range and depth of volunteers' contribution to specialist paediatric palliative care services and will help to provide a basis for future research, which could inform expansion of volunteers' roles.	None	Volunteers entirely ran some services, notably complementary therapy and pastoral/faith-based care. Complementary therapists, school teachers and spiritual care workers most commonly volunteered their professional skills.		
Carrero Planes 2015	The tool consists of 17 items. Sixteen of them have a Likert-type scale response and one has a "free association words" format response. The exploratory content analysis allows us to identify four dimensions in the meaning of the AD at EoL: "Significant Meeting, Redemption, Resilience and Resignification". Conclusions. Four areas of psychosocial/spiritual intervention are proposed in order to detect existential-meaning sources during the AD. Practical implications for preventing existential suffering, demoralization syndrome and pathological bereavement at the EoL are [..?].	INTRODUCTION: 'Desde esta aproximación se enfatiza la dimensión existencial y espiritual, para dar sentido a "quién soy yo", "para qué estoy aquí" y "qué es lo que puedo esperar de este momento", en situaciones vitales de EA' (enfermedad avanzada). Translation: 'In this approach, the existential and spiritual dimensions are emphasized, in order to give meaning to "who I am", "why I am here" and "what can I hope from this moment" in situations of advanced illness' || DISCUSSION: 'Casi de un modo inevitable la persona se da cuenta que existe una naturaleza más allá de los límites físicos e individuales'. Translation: 'Almost inevitably, the person realizes that there is a nature beyond the physical and invidual limits.'	The exploratory content analysis allows us to identify four dimensions in the meaning of the AD at EoL: "Significant Meeting, Redemption, Resilience and Resignification".		
Ettema 2015	This study shows that the spiritual dimension is only embedded to a limited extent in the PCTs. Most respondents are of the opinion that their team lacks expertise in spiritual care, the majority of the consultants do not receive regular training in dealing with the spiritual dimension, and many plans for education and training are in a preliminary stage. These limitations, however, go together with a clear desire for education and training in dealing with the spiritual dimension. Although most teams do not clearly distinguish between psychological, social and spiritual competences, the healthcare chaplain is most often mentioned as the expert in the field of spiritual care.	INTRODUCTION: Patients, care providers and researchers find it hard to define spirituality, to list its characteristics and to effectively communicate about it in the daily practice of health care. In addition, due to the broad diversity in world views in the Netherlands, caregoivers may prefer to employ other terms instead of spirituality, such as faith, phylosophy of life, giving meaning, life story, world view, and religious life. Furthermore, caregivers may feel uncomfortable discussing spiritual questions with their patients || DISCUSSION: spirituality is related to the often unconscious way in which individuals 'experience, express and/or seek meaning, purpose and transcendence.		Nearly all respondents (96%) agree that it is the Palliaive Care Team's (PCT's) responsibility to deal with questions about the spiritual dimension. Most of them (84%) think the team should pay attention to the spiritual dimension even when the advice seeking professional does not mention it. Others (12%) take the view that the team should only pay attention to the spiritual dimension when the advice seeking professional asks for it. One respondent considered attention to the spiritual dimension and going into specific questions concerning this dimension not a responsibility of the team.	The need for education and training for PCT memberd in dealing with the spiritual dimension. Establishing an inventory of visions on what good spiritual care consists of, so that consultants become acquainted with barriers to spiritual care and become skilled in dealing with the hidden hopes and fears of the patient. The chaplain should be a permanent team member who is on call.

Kögler 2015	High correlations between mindfulness and mental distress (r = - 0.51, p , 0.001) as well as life satisfaction (r = 0.52, p , 0.001) were found. Mindfulness was a significant predictor of improvement in psychological distress, meaning in life and quality of life three months after the intervention. The EBT effects were partly mediated by mindfulness. Significance of results: Mindfulness seems to be a promising concept in supporting informal caregivers of PC patients. Further research is needed to identify the required format and intensity of mindfulness practice necessary for improvement.	INTRODUCTION: Mindfulness, is originally a Buddhist concept and has been described as: "the awareness that emerges through paying attention on purpose, in the present moment and non-judgementally to the unfolding of experiencce moment by moment"	Existential Behavioral Therapy (EBT) has been developed to support informal caregivers facing the imminent or recent loss of a family member in a palliative care (PC) setting. Mindfulness training was a core element of the intervention. The EBT groups (six sessions,m aximum of 10 participants/group, 22 hours in total) were led by trained psychotherapists. Information on mindfulness was given during the first meetings. Every session included formal mindfulness practice (e.g., following one's breath while noticing and letting go of all thoughts, feelings and sensations) for at least 15 minutes. Participants received CD recordings with mindfulness exercises and were encouraged to practice at home at least twice a day for a minimum of 10 minutes. Furthermore, informal mindfulness (i.e., performing daily activities mindfully, e.g., brushing teeth, preparing meals) was practiced.	Short and longterm effects of this intervention on quality of life (QoL) and psychological distress have been found up to 12 months after treatment. Mindfulness in informal caregivers of PCpatients was significantly correlated with higher QoL, life satisfaction, the experience of meaning, and lower psychological distress. Regarding the facets of mindfulness, a negative correlation between psychological distress and "Describing/ labeling with words," "Acting with awareness," "Non-reactivity to inner experience," and "Non-judging of experience" was found. These attitudes may help relatives to cope with their experiences. No correlations were found with "Observing": this might be explained by the assumption that observation of internal experiences alone is not adaptive without the accepting attitude cultivated in meditation. Mindfulness has both been described as state and trait. With regard to trait, dispositional mindfulness (T1) was a significant predictor of adjustment for all relatives in both groups. The effect was most significant at the 3-months follow-up (T1/T3). Regarding changes of mindfulness (as a state) following the EBT intervention, small but significant effects were found. Long-term effects of the EBT intervention on depression and QoL appeared to be partly mediated by indfulness. Participants who indicated the highest levels of formal practice had a stronger increase in mindfulness and meaning in life; those with the highest levels of informal practice showed stronger improvements in QoL, life satisfaction, and meaning in life.	Further research on underlying mechanisms is needed, concerning the format, intensity and type of practice which are most effective in improving well-being und reduce psychological distress. Mindfulness training may be a promising concept for psychosocial support in palliative care.
Llewellyn 2015	(1) HCP conceptualised spirituality as highly individualised searches for meaning, hope and connectedness to self, others and the world. They saw spirituality within a developmental context. (2) HCP described spiritual concerns that were tied to their own conceptualisations of spirituality, centring on ideas of loss, including loss of hope or meaning. (3) HCP approached spiritual concerns of CYP and families by 'being there' and supporting spiritual enquiry. (4) Challenges to their work included managing hopes of CYP and families in the face of poor prognoses, discussions about miracles and issues with their own faith. Spiritual care was seen as different to other areas of care which HCP felt had a greater prescription in delivery.	INTRODUCTION: spirituality provides a framework to make sense of and find meaning in experiences; use the working definition of the European Association of Palliative Care || RESULTS: HCP conceptualised spirituality for both adults and CYP as searches for meaning and hope. Spirituality was also thought to support relationships between people and between individuals and the transcendent, and tied closely to self-identity. There was strong agreement among HCP that spirituality is at once a broad and highly individualised construct, which might include orthodox as well as unorthodox beliefs. They recognised how spiritual beliefs might assume particular significance during illness and premature death as a way of making sense of tragedy and in guiding approaches to decisions concerning medical treatment. HCP saw children to be at the beginning of their spiritual enquiries, at first influenced largely by their family and cultural norms and characterised by magical thinking. However, as they mature and grow older it was thought that their beliefs might become more individualised	(3) HCP approached spiritual concerns of CYP and families by 'being there' and supporting spiritual enquiry. (4) Challenges to their work included managing hopes of CYP and families in the face of poor prognoses, discussions about miracles and issues with their own faith. Spiritual care was seen as different to other areas of care which HCP felt had a greater prescription in delivery.		

McTiernan 2015	Three master themes emerged from the analysis: the personal impact of diagnosis, the struggle in adjusting to change, and dying in context. The results revealed that participants were still living while simultaneously dying. Interestingly, participants did not ascribe new meaning to their lives. The terminal illness was understood within the framework of the life that had existed before diagnosis. They strove to maintain their normal routines and continued to undertake meaningful activities. Management of unfinished business and creation of a legacy were salient tasks. Social withdrawal was not present; rather, participants engaged in emotional labor to sustain valued roles. However, we found that within the public domain there is a paucity of education and discourse supporting individuals at the end of life. The hospice was noted as an important external resource. Each participant experienced a unique dying process that reflected their context. Significance of Results: Healthcare professionals need to recognize the subjectivity of the dying process. Dying individuals require support and options to maintain their personhood.	RESULTS: When individuals engage with their inner process, the meaning of the illness becomes less overwhelming.	In-depth interventions reconnect individuals with meaningful spects of life. Individuals may pursue writing, music, life review, psychotherapy, and therapeutic toch.	Results show that patients percieve diagnosis as a biographical disruption resulting in shock. Terminal diagnosis initiates a life review. Patients search to find a reason for the illness. Three participants reported improved quality of life. Participants used numerous methods of coping, incluing drawing on beliefs. Religious beliefs can construct meaning and reduce suffering. Three participants believed in their self-efficacy, which links with improved coping. Patients focussed on living, not dying. Participants set goals, such tasks maintain selfesteem, protect against death anxiety and facilitate attendng to controlable aspects of life. Humor was also an important coping mechanism, concentration on the present and completing unfinished business. Creating legacies. External sources centred on cure, hope, quality and lengthening of life.	However, we found that within the public domain there is a paucity of education and discourse supporting individuals at the end of life. The hospice was noted as an important external resource. Each participant experienced a unique dying process that reflected their context. : Healthcare professionals need to recognize the subjectivity of the dying process. Dying individuals require support and options to maintain their personhood.
Paal 2015	Key points of the article: a) Palliative care professionals and volunteers need to be trained in recognising spiritual issues and in delivering spiritual care; b) The education sub group of the EAPC Task Force on Spiritual Care in Palliative Care has surveyed EAPC members to identify spiritual care training courses, current or planned. Data were gathered regarding 36 courses in 14 countries, mostly in Europe; c) The education sub group makes recommendations regarding spiritual care training, encourages EAPC members to invest in such training, and welcomes further responses to its survey, as this will allow it to extend its database of training courses.	RESULTS: 81% of all trainings used the EAPC working definition of spirituality		Key points of the article: a) Palliative care professionals and volunteers need to be trained in recognising spiritual issues and in delivering spiritual care; b) The education sub group of the EAPC Task Force on Spiritual Care in Palliative Care has surveyed EAPC members to identify spiritual care training courses, current or planned. Data were gathered regarding 36 courses in 14 countries, mostly in Europe; c) The education sub group makes recommendations regarding spiritual care training, encourages EAPC members to invest in such training, and welcomes further responses to its survey, as this will allow it to extend its database of training courses.	
Papadaniel 2015	We have observed that many of the interviewees still vividly remember the small gestures and words of a nurse or a physician perceived as “very human”. These short narratives show how close relatives of very ill patients may develop a sudden attachment to people who have behaved as if they had truly, immediately understood their ordeal. Humanity is thus the keyword of this article, in which we will analyse its ambiguous meaning through the lens of individual’s everyday experiences	None	These short narratives show how close relatives of very ill patients may develop a sudden attachment to people who have behaved as if they had truly, immediately understood their ordeal. Humanity is thus the keyword of this article, in which we will analyse its ambiguous meaning through the lens of individual’s everyday experiences	We have observed that many of the interviewees still vividly remember the small gestures and words of a nurse or a physician perceived as “very human”.	
Ross 2015	Participants were struggling with spiritual/existential concerns alongside the physical and emotional challenges of their illness. These related to: love/belonging; hope; coping; meaning/purpose; faith/belief; and the future. As a patient's condition deteriorated, the emphasis shifted from 'fighting' the illness to making the most of the time left. Spiritual concerns could have been addressed by: having someone to talk to; supporting carers; and staff showing sensitivity/taking care to foster hope. A spiritual support home visiting service would be valued.	INTRODUCTION: There is evidence that the psychological and spiritual domains are distinct but related. Whereas the psychological domain is concerned with affect, cog- nition, self-esteem and body image, the spiritual domain is concerned with spiritual/religious/personal beliefs, connection, meaning, wholeness and spiritual strength (O’Connell & Skevington 2010). Examination of the range of definitions of spirituality across disciplines involving diverse groups reveals common elements: hope and strength; trust; meaning and purpose; forgiveness; love and relationships; belief and faith; and peoples’ values, morality, creativity and self expression (RCN 2011, McSherry & Ross 2012) || RESULTS: Spiritual issues were significant for the sample with all 16 patients / carers struggling with spiritual/existential concerns. These related to six main themes which corresponded with the key concepts of spirituality identi- fied in the literature (e.g. RCN 2011): love & belonging, hope & coping, meaning & purpose, faith & belief & existential issues,	Spiritual concerns could have been addressed by: having someone to talk to; supporting carers; and staff showing sensitivity/taking care to foster hope. A spiritual support home visiting service would be valued.		

Rudilla 2015	In order to achieve this objective, a three-week intervention was carried out with 131 home care and hospitalized patients. The mean age was 70.61 (SD = 11.17); 51.1% were men. Spirituality was assessed before and after the intervention, and a multivariate analysis of variance (MANOVA) was used to study the differences between these two moments, together with follow-up ANOVAs. Results indicated a positive effect, with a large effect size, $F(3, 110) = 31.266$, $p < .001$, $\eta2 = .460$.	INTRODUCTION: Las necesidades espirituales, cuando son elaboradas de forma efectiva, ayudarán a la persona al final de la vida a encontrar signifi-cado, mantener la esperanza y aceptar la muerte (Corr y Corr, 2000). Translation: Spiritual needs, when elaborated effectively, will help the person at the end of life to find meaning, maintain hope and accept death (Corr and Corr, 2000) || METHOD: Estas ocho preguntas evalúan un factor general de espiritualidad, que a su vez se compone de tres dimensiones de espiritualidad: espiritualidad intrapersonal (las relaciones con uno mismo, la necesidad de sentido y coherencia), interpersonal (las relaciones con otros, la armonía con las relaciones con las personas que más preocupan y la necesidad de ser amados y amar) y transpersonal (conciencia de pertinencia a una dimensión transcendente, confianza y esperanza y/o legado que se deja) (Galia- na, Oliver, Gomis Barbero y Benito, 2014). Translation: These eight questions evaluate a general factor of spirituality, which in turn consists of three dimensions of spirituality: intrapersonal spirituality (relationships with oneself, the need for meaning and coherence), interpersonal (relationships with others, harmony with relationships with the people who are most concerned and the need to be loved and loved) and transpersonal (awareness of relevance to a transcendent dimension, trust and hope and / or legacy left) (Galiana, Oliver, Gomis Barbero and Benito, 2014).		Results indicated a positive effect, with a large effect size, $F(3, 110) = 31.266$, $p < .001$, $\eta2 = .460$.	
Thomas 2015	Analysis of the twenty-five interviews revealed difficulties in finding a language that both expresses spirituality and the 'soft' descriptions of what happens in spiritual care and satisfies the 'inquisition' of outcome oriented management.	RESULTS: difficulties in finding a language that expresses spirituality.	difficulties in finding a language that expresses spirituality.		
Tornøe 2015a	The mobile teaching team taught care workers to identify spiritual and existential suffering, initiate existential and spiritual conversations and convey consolation through active presencing and silence. The team members transferred their personal spiritual and existential care knowledge through situated "bedside teaching" and reflective dialogues. "The mobile teaching team perceived that the care workers benefitted from the situated teaching because they observed that care workers became more courageous in addressing dying patients' spiritual and existential suffering. Discussion: Educational research supports these results. Studies show that efficient workplace teaching schemes allowexpert practitioners to teach staff to integrate several different knowledge forms and skills, applying a holistic knowledge approach. One of the features of workplace learning is that expert nurses are able to guide novices through the complexities of practice. Situated learning is therefore central for becoming proficient. Conclusions: Situated bedside teaching provided by expert mobile hospice nurses may be an efficient way to develop care workers' courage and competency to provide spiritual and existential end-of-life-care. Further research is recommended on the use of mobile expert nurse teaching teams to improve nursing competency in the primary health care sector.	None	As there seems to be no single agreed definition of spiritual care in research literature, the term is open for interpretation. This study has therefore adopted a pragmatic and functionalist epistemological point of departure since it is targeted at the practical implications of the mobile teaching team;'s experience, rather than the onthological questions telated to the conceptual framework of spiritual care.	The mobile teaching team taught care workers to identify spiritual and existential suffering, initiate existential and spiritual conversations and convey consolation through active presencing and silence. The team members transferred their personal spiritual and existential care knowledge through situated "bedside teaching" and reflective dialogues. The mobile teaching team perceived that the care workers benefitted from the situated teaching because they observed that care workers became more courageous in addressing dying patients' spiritual and existential suffering.	The practice of the mobile teaching team taught care workers to identify spiritual and existential suffering, initiate existential and spiritual conversations and convey consolation through active presencing and silence. The team members transferred their personal spiritual and existential care knowledge through situated "bedside teaching" and reflective dialogues. "The mobile teaching team perceived that the care workers benefitted from the situated teaching because they observed that care workers became more courageous in addressing dying patients' spiritual and existential suffering.

Tornøe 2015b	The nurses felt that it was challenging to uncover dying patients' spiritual and existential suffering, because it usually emerged as elusive entanglements of physical, emotional, relational, spiritual and existential pain. The nurses' spiritual and existential care interventions were aimed at facilitating a peaceful and harmonious death. The nurses strove to help patients accept dying, settle practical affairs and achieve reconciliation with their past, their loved ones and with God. The nurses experienced that they had been able to convey consolation when they had managed to help patients to find peace and reconciliation in the final stages of dying. This was experienced as rewarding and fulfilling. The nurses experienced that it was emotionally challenging to be unable to relieve dying patients' spiritual and existential anguish, because it activated feelings of professional helplessness and shortcomings. Conclusions: Although spiritual and existential suffering at the end of life cannot be totally alleviated, nurses may ease some of the existential and spiritual loneliness of dying by standing with their patients in their suffering. Further research (qualitative as well as quantitative) is needed to uncover how nurses provide spiritual and existential care for dying patients in everyday practice. Such research is an important and valuable knowledge supplement to theoretical studies in this field.	INTRODUCTION: The nursing literature interprets and applies the terms "spiritual" and "existential" care in different ways, which suggests that these terms are open to interpretation. Sev- eral scholars support this view [6, 23, 26–29] and ac- cording to them, there seems to be no single agreed definition on spiritual and/or existential care in nursing literature.	The nurses felt that it was challenging to uncover dying patients' spiritual and existential suffering, because it usually emerged as elusive entanglements of physical, emotional, relational, spiritual and existential pain. The nurses' spiritual and existential care interventions were aimed at facilitating a peaceful and harmonious death. The nurses strove to help patients accept dying, settle practical affairs and achieve reconciliation with their past, their loved ones and with God. The nurses experienced that they had been able to convey consolation when they had managed to help patients to find peace and reconciliation in the final stages of dying. This was experienced as rewarding and fulfilling. The nurses experienced that it was emotionally challenging to be unable to relieve dying patients' spiritual and existential anguish, because it activated feelings of professional helplessness and shortcomings.		
Vermandere 2015	The Ars Moriendi Model (AMM) was perceived as valuable. Many patients shared their wishes and expectations about the end of life. Most HCPs said they felt that the patient-provider relationship had been strengthened as a result of the spiritual assessment. Almost all assessments raised new issues; however, many dyads had informally discussed spiritual issues before. Conclusions: The current study suggests that HCPs believe that the AMM is a useful spiritual assessment tool. Guided by the model, HCPs can gather information about the context, life story, and meaningful connections of patients, which enables them to facilitate person-centered care. Implications for Nursing: The AMM appears to be an important tool for spiritual assessment that can offer more insight into patients' spirituality and help nurses to establish person-centered end-of-life care.	INTRODUCTION: The open structure and diamond shape of the model provide flexibility and spontaneity in the communication about spirituality. The questions of the model are formulated in spoken language (Leget, 2007), and five tension fields are presented (i.i. autonomy, pain control, attachment and relations, guilt and evil, and the meaning of lfe).		The AMM was perceived as valuable. Many patients shared their wishes and expectations about the end of life. Most HCPs said they felt that the patient-provider relationship had been strengthened as a result of the spiritual assessment. Almost all assessments raised new issues; however, many dyads had informally discussed spiritual issues before.	
Woolf 2015	Participants' ill bodies had become obstacles to them expressing their sense of self. The safe therapeutic conditions, gentle movement, music, words and props in DMP (Dance Movement Psychotherapy) enabled them to express interconnected physical and emotional pain and loss of sense of self. DMP enabled them to reintegrate with their estranged bodies, releasing tension by expressing themselves as a whole person.	None	The safe therapeutic conditions, gentle movement, music, words and props in DMP (Dance Movement Psychotherapy) enabled them to express interconnected physical and emotional pain and loss of sense of self. DMP enabled them to reintegrate with their estranged bodies, releasing tension by expressing themselves as a whole person.	The safe therapeutic conditions, gentle movement, music, words and props in DMP (Dance Movement Psychotherapy) enabled them to express interconnected physical and emotional pain and loss of sense of self. DMP enabled them to reintegrate with their estranged bodies, releasing tension by expressing themselves as a whole person.	
Bekkema 2016	Two dimensions of care relationships were found, of which one was 'being there': ill people need people who are there for him or her practically, as wel as emotionally, socially and spiritually.	None	Two dimensions of care relationships were found, of which one was 'being there': ill people need people who are there for him or her practically, as wel as emotionally, socially and spiritually.		
Brinkman-Stoppelenburg 2016	Seventy-seven percent of all participating hospitals had a palliative care team. (..) The most common disciplines were nurses (72 %), and nurse practitioners (54 %), physicians specialized in internal medicine (90 %), or anaesthesiology (75 %), and spiritual caregivers (65 %).	None	Geen beschrijving van spirituele zorg	In 65 % of the palliative care teams, spiritual caregivers took part.	
Dones Sánchez 2016	The sample included a high percentage of women, mostly nurses and psychologists. Of those, 94.2% considered that spiritual care was part of their professional role, but only 57.6% considered themselves competent for this task. Less than half (41.9%) said they had a specific person for spiritual care on their teams, but only a 45% of them considered specifically spiritual aspects of teams.	None	Of those, 94.2% considered that spiritual care was part of their professional role, but only 57.6% considered themselves competent for this task. Less than half (41.9%) said they had a specific person for spiritual care on their teams, but only a 45% of them considered specifically spiritual aspects of teams.		57.6% considered themselves competent for the task of spiritual care
Evenblij 2016	Thirty-six percent of nurses had experience with providing palliative care to psychiatric patients with physical co-morbidity in the past 2 years. Of all patients, 63% received physical care before death, 46% psychosocial care and 33% spiritual care. In 91% of all cases, care was provided by multidisciplinary teams. Patient characteristics and little attention to palliative care were barriers for timely and adequate palliative care.	None	Of all patients, 63% received physical care before death, 46% psychosocial care and 33% spiritual care. In 91% of all cases, care was provided by multidisciplinary teams.		

Goodhead 2016	Participants described their experiences of ordination training and how helpful this had been for their work among Christian communities. Respondents were invited to discuss their knowledge of and involvement with palliative care services. Each interviewee also accounted for their understanding of pastoral care and spiritual care and considered whether any differences existed between these terms and, if so, what they were. Overall, clergy lacked any detailed formal training and had little experience of working with or relating to palliative care providers. Recommendations are made to improve educational opportunities and working relationships.	None	The need to identify and respond to the spiritual needs of patients with incurable, progressive disease and their families is enshrined in palliative care policy guidance globally	All participants had undertaken classroom-based training, involving university-validated courses and in-house training. Pastoral care education was not perceived as a priority of training. Placements were integral to training, usually in areas of personal interest or a longer term hospital placement. Some placements had impact. Participants' experience of continuing education was mixed. Awareness of hospice and palliative care services was also mixed. Clergy described the relationship between themselves and the individual as central to pastoral care. The theme of 'journey' for the recipient of pastoral care was present in many interviews and was described with eschatological implications. Pastoral care was further described using terms such as 'caretaker', 'shepherds', 'sheep', 'flock', 'pasture', 'servant' and 'pastor'. Participants struggled to describe their perception of spiritual care, frequently conflating spiritual care with pastoral care.	Overall, clergy lacked any detailed formal training and had little experience of working with or relating to palliative care providers. Dialogue between clergy and healthcare providers is lacking. Recommendations are made to improve educational opportunities and working relationships.
Gratz 2016	All training programs included self-reflection on personal spirituality as obligatory. The definitions of spirituality used in programs differ considerably. The task of defining training objectives is randomly delegated to a supervisor, a trainer, or to the governing organization. More than half the institutions work in conjunction with an external trainer. These external trainers frequently have professional backgrounds in pastoral care/theology and/or in hospice/palliative care. While spiritual care receives great attention, the specific tasks it entails are rarely discussed.	INTRODUCTION: The indeterminate character of spirituality is an essential element of its description or definition, because spirituality is "precisely—and exclusively— that which the patient deems to be spirituality". || RESULTS: Some 86.5% (n ¼ 45) of respondents answered affirmatively to the question of whether the course discussed the meaning of the term "spirituality." In light of the many significances for the meaning of "spirituality" that can be found in the pertinent literature, the courses work with a diverse set of definitions for the term.	No clear definition: [Results] "They attributed a relatively high level of importance to spiritual care, and deemed it equally important to instruct hospice volunteers in the specific tasks connected to spiritual care".	All training programs included self-reflection on personal spirituality as obligatory. The definitions of spirituality used in programs differ considerably. The task of defining training objectives is randomly delegated to a supervisor, a trainer, or to the governing organization. More than half the institutions work in conjunction with an external trainer. These external trainers frequently have professional backgrounds in pastoral care/theology and/or in hospice/palliative care.Other topics: the need for continued training, relations with church-based organizations, an with pastoral care.	the integration of spirituality and spiritual care into a concept for training volunteers remains a challenging task. It requires a shared and accepted basis that elucidates the meaning of the term "spirituality" for the purposes of training. However, a relevant understanding of spirituality itself is accessible only through encounters with patients and family members. This approach firmly rejects intentions to impose external standards of spirituality on recipients of hospice care. It also suggests a form of spiritual care that achieves expert management of the aspects of presence, listening, perception, acceptance, respect, and reaction.
Kienle 2016	The doctors integrated conventional and holistic cancer concepts. Their treatments aimed at both tumor and symptom control and at strengthening the patient on different levels: living with the disease, overcoming the disease, enabling emotional and cognitive development, and addressing spiritual or transcendental issues according to the patient's wishes and initiatives.	None	addressing spiritual or transcendental issues according to the patient's wishes and initiatives.		
Kruizinga 2016	Although the spiritual counselors were experiencing struggles with structure and iPad, they were immediately willing to work with the new structured method as they expected the visibility and professionalization of their profession to improve. In this process, they experienced a need to adapt to a certain role while working with the new method and described how the identities of the profession were challenged.	None			Although the spiritual counselors were experiencing struggles with structure and iPad, they were immediately willing to work with the new structured method as they expected the visibility and professionalization of their profession to improve. In this process, they experienced a need to adapt to a certain role while working with the new method and described how the identities of the profession were challenged.
Nolan 2016		None	[Intro] "An active process of finding people who need spiritual care, identifying the nature of the need and responding to the need through theological reflection and the sharing of spiritual practices". [Disc] For me, the idea of being present, or being with, is core to spiritual care. .. But I try also to be open to what I represent, or evoke as a chaplain.	No concrete 'result' in this case study. "Such a approach did not reduce my chaplaincy to a form of humanistic psychotherapy, nor did it induce tension in me as one who is both a chaplain and psychotherapist.	The evolving healthcare context demands chaplains demonstrate the value of chaplaincy work with people who regard themselves as non-religious, in this case people who regard themselves as non-religious, in this case whose religion is being secularized, but whose secularism is touched by the sacred.

Noome 2016	The focus group interviews resulted in five themes: (i) awareness of ICU nurses, (ii) communication, (iii) nursing interventions, (iv) multidisciplinary care and (v) education. In total, twenty recommendations were formulated.	INTRODUCTION: "a person's search for or expression of his or her connection to a greater and meaningful contect". "The search for attention to the ultimate meaning and purpose in life, often involving a relationship with the transcendent' and focused on the constuct of 'being at peace'"	No definition of spiritual care.	The focus group interviews resulted in five themes: (i) awareness of ICU nurses, (ii) communication, (iii) nursing interventions, (iv) multidisciplinary care and (v) education. In total, twenty recommendations were formulated.	Spiritual care should be standard care, by adding spiritual needs more specifically into the data of the patient's history. Nurses should know themselves and avoid judgements an prejudices. They should be aware of their own background, through education and reflection. They should identify their own communication style and compre it with those of patient and family. Spiritual care should be multidisciplinary. Nurses should provide standard spiritual care. requirements: training on all these skills.
Olsman 2016	When participants spoke about hope, they referred to power and empowerment, like the powerful bonding of hope between patients and physicians. They also associated hope with the loss of hope and suffering. Several participating healthcare professionals tried to balance both sides, which involved acknowledgment of hope and suffering. Hope and power were reflected in the ethical concept of empowerment, whereas suffering and the loss of hope were reflected in the ethical concept of compassion.	None	Several participating healthcare professionals tried to balance both sides, which involved acknowledgment of hope and suffering. Hope and power were reflected in the ethical concept of empowerment, whereas suffering and the loss of hope were reflected in the ethical concept of compassion.		
Ortega Galán 2016	It was found that the spiritual dimension is weakly integrated into the nursing image of the care in the process of dying and, therefore, little valued as an important element in the support of the sick person in the terminal phase.	None	It was found that the spiritual dimension is weakly integrated into the nursing image of the care in the process of dying and, therefore, little valued as an important element in the support of the sick person in the terminal phase.		
Rufino Castro 2016	During the study period the palliative care team attended 276 new patients, of whom 86 (31.2%) expressed 119 comments spiritual and 102 spiritual needs. Nurses were the professionals who identified more spiritual comments (41.2%) and the hospital consultation the location where most were recorded (48.8%). In relation to the spiritual domains, 93.7% of the 86 patients expressed expressions that were included in the domain of intrapersonal, 61.7% made reference to the interpersonal domain and 26.8% to the transpersonal. Within the intrapersonal domain highlighted that a 41.8% of the patients with spiritual comments referred to hopelessness- desire for hastened death. Respect to the spiritual needs the highest percentages corresponded to the need to lifetime re-examination (22% of patients) and the need to look for meaning to existence (25.5% of patients).	None	During the study period the palliative care team attended 276 new patients, of whom 86 (31.2%) expressed 119 comments spiritual and 102 spiritual needs. Nurses were the professionals who identified more spiritual comments (41.2%) and the hospital consultation the location where most were recorded (48.8%). In relation to the spiritual domains, 93.7% of the 86 patients expressed expressions that were included in the domain of intrapersonal, 61.7% made reference to the interpersonal domain and 26.8% to the transpersonal. Within the intrapersonal domain highlighted that a 41.8% of the patients with spiritual comments referred to hopelessness- desire for hastened death. Respect to the spiritual needs the highest percentages corresponded to the need to lifetime re-examination (22% of patients) and the need to look for meaning to existence (25.5% of patients).		
Serra Vila 2016	Preferred techniques are singing (47%) and playing or improvising with instruments (46%). Emotional support (9.1/10) and relaxation (9/10) are the main achievements obtained. Qualitative responses are distributed into three categories: Verbalization of the illness, benefits of the MT, and suggestions for the program. The most emphasized benefits are: perception of support, active family participation, relaxation and well-being, simplification of the communication, mood improvement, different perception of time passing and connection with spirituality. The overall evaluation of the MT programme received a mean score of 9.43/10.	None	Singing; Playing or improvising with instruments; Verbalization of the illness	(Quantitative?): Emotional support (9.1/10) and relaxation (9/10) are the main achievements obtained; (Qualitative?): The most emphasized benefits are: perception of support, active family participation, relaxation and well-being, simplification of the communication, mood improvement, different perception of time passing and connection with spirituality.	
Søfting 2016	Our study indicates that it was very important for the children to be included in the rituals and accordingly be recognized as grievers alongside adults. Being included contributes to legitimating their status as a "full" member of the family system, with an equal status to adult grievers in an important and vulnerable phase of the family's life. The children were pleased that they through ritual performances were given the opportunity to "see for themselves," both in order to better comprehend and accept the reality of the loss and to take farewell with their loved ones.	None	To include children in rituals and accordingly be recognized as grievers alongside adults	Rituals: to better comprehend and accept the reality of the loss and to take farewell with their loved ones.	

Steenfeldt 2016	Four caring themes emerged from data analysis: recognized as an individual human being; caring as doing and being; caring for the whole body; and spaces of caring. Spiritual care was understood as providing whole-body experiences, respecting the patient, and involving the other person.	There are different kind of spiritual needs; one is the need for being perceived as an individual being. Some existential needs are related to the physical changes of the body and the consequent changes in the patients' self-image. Other needs are related to finding meaning and to experience courage and hope.	Spiritual care was understood as providing whole-body experiences, respecting the patient, and involving the other person.		
Stockle 2016	44/102 (43,1 %) of eligible informal caregivers agreed to participate in the study. Due to attrition of 13 caregivers (attrition rate: 29,5 %), 31 caregivers were included in the trial. Self-rated usefulness showed sufficiant results for all but one individual aspect. Frequency of implementing therapeutic elements showed wide inter-item as well as inter-participant ranges and decreased over the study period. All participants completed both sessions. Return rates of the questionnaires were within the expected range. According to the interviews, the intervention was associated with several participant-identified benefits. No severe adverse effects were observed.	None	Existential Behavioral Therapy (EBT) is based on existential behavioral psychology, and the 'third wave' of behavioral herapy. Concepts such as mindfulness, metacognition, acceptance, personal values, meaning in life and spirituality were integrated in the 'third wave' therapies. Existential Behavioral Therapy (EBT) has been developed to support informal caregivers facing the imminent or recent loss of a family member in a palliative care (PC) setting. Mindfulness training was a core element of the intervention.	44/102 (43,1 %) of eligible informal caregivers agreed to participate in the study. Due to attrition of 13 caregivers (attrition rate: 29,5 %), 31 caregivers were included in the trial. Self-rated usefulness showed sufficiant results for all but one individual aspect. Frequency of implementing therapeutic elements showed wide inter-item as well as inter-participant ranges and decreased over the study period. According to the interviews, the intervention was associated with several participant-identified benefits. No severe adverse effects were observed.	The intervention showed to be acceptible in most aspects, future research is needed to focus on adjustments with regard to the less well recieved aspects of the intervention.
van Lancker 2016	On average, 5·15 disciplines were consulted per patient. Few patients were referred to a palliative support team, spiritual consultant or psychologist.	One spiritual item of the Assessment Symptoms Palliative Elderly: 'Experience life as not meaningful'	Few patients were referred to a palliative support team, spiritual consultant or psychologist.		
Vermandere 2016	Registered nurses and general practitioners approached eligible patients with an incurable, life-threatening disease for study participation. Health-care providers allocated to the intervention arm of the study took a spiritual history on the basis of the ars moriendi model. Health-care providers in the control arm provided care as usual. Patient-reported outcomes on spiritual well-being, quality of life, pain, and patient-provider trust were assessed at two points in time. RESULTS: A total of 245 health-care providers participated in the study (204 nurses and 41 physicians). In all, 49 patient-provider dyads completed the entire study protocol. The median age of the patients was 75 years (range: 41-95 years), and 55% of the patients were female. There were no significant differences at any point in time in the scores on spiritual well-being, quality of life, pain, or patient-provider trust between the intervention and the control group.	We hypothesized that the ars moriendi model (AMM) might be a feasible tool for spiritual history taking in palliative care (ref) This model has it roots in the Middle Ages and sketches five temptations that present themselves to the dying person: the loss of faith, the loss of one's confidence in salvation, the hanging on to temporal affairs, the inability to deal with pain and suffering (...). Leget (ref) updated this model to modern culture and challenges. (See Vermandere 2015 in this Appendix).	We hypothesized that the ars moriendi model might be a feasable tool for spiritual history taking in palliative care.	There were no significant differences at any point in time in the scores on spiritual well-being, quality of life, pain, or patient-provider trust between the intervention and the control group.	More research is needed to better understand the constructs of spirituality that are relevant for the assessment of a patients' current spiritual care. We need to develop instruments that accurately assess the effectiveness of spiritual interventions in palliative care populations.
Willig 2016	Object elicitation was used to assist data collection by facilitating participants' reflections on the quality and texture of their lived experience. Participants were invited to select objects that held special meaning for them during the current phase of their lives and to reflect on their relationship with these objects during a research interview. This paper reflects upon the opportunities and challenges inherent in the use of object elicitation. These include the method's ability to prompt unrehearsed, in-the-moment reflections about what it means to be "living with dying" as well as to shed light on participants' sense of who they can be during this final phase of their lives. At the same time, the focus on objects can result in the imposition of an object-led structure on the interviews and a consequent failure to follow up on aspects of participants' accounts that transcend their relationship with the objects they brought. A further challenge resides in the temptation to look for meaning in the objects themselves rather than in the participants' use of, and relationship with, the objects. The paper formulates guidance on the use of object elicitation.	None	Participants were invited to select objects that held special meaning for them during the current phase of their lives and to reflect on their relationship with these objects during a research interview.	This paper reflects upon the opportunities and challenges inherent in the use of object elicitation. These include the method's ability to prompt unrehearsed, in-the-moment reflections about what it means to be "living with dying" as well as to shed light on participants' sense of who they can be during this final phase of their lives.	The paper formulates guidance on the use of object elicitation. The focus on objects can result in the imposition of an object-led structure on the interviews and a consequent failure to follow up on aspects of participants' accounts that transcend their relationship with the objects they brought. A further challenge resides in the temptation to look for meaning in the objects themselves rather than in the participants' use of, and relationship with, the objects. The paper formulates guidance on the use of object elicitation.
Zenz 2016	The wish for hospice treatment (44.8%) or spiritual care (39.3%) was less frequent.	None	The wish for hospice treatment (44.8%) or spiritual care (39.3%) was less frequent.		
De Graaf 2017	The physical dimension was most prevalent in daily care, reflecting the patients' primary expressed priority at admission and the nurses' and physicians' primary focus. The psychological, social and spiritual dimensions were less frequently described. Assessment tools were used systematically by 4/12 hospices. Facilitators identified were interdisciplinary collaboration, implemented methods of clinical reasoning and structures.	METHOD: the spiritual dimension was defined as information about religion, meaning and existential well-being.	The psychological, social and spiritual dimensions were less frequently described. Assessment tools were used systematically by 4/12 hospices.		

Giezendanner 2017	Ninety-nine percent of GPs considered the recognition and treatment of pain as important, 86% felt confident about it. Few GPs felt confident in cultural (16%), spiritual (38%) and legal end-of-life competencies such as responding to patients seeking assisted suicide (35%) although more than half of the respondents regarded these competencies as important. Most frequent reasons to refer terminally ill patients to a specialist were lack of time (30%), better training of specialists (23%) and end-of-life care being incompatible with other duties (19%). In multiple regression analyses, confidence in end-of-life care was positively associated with GPs' age, practice size, home visits and palliative training.	DISCUSSION: Spirituality is a major domain of palliative care training to ensure that patients can find meaning and hope even in the last period of their life	Few GPs felt confident in cultural (16%), spiritual (38%) and legal end-of-life competencies such as responding to patients seeking assisted suicide (35%) although more than half of the respondents regarded these competencies as important. Most frequent reasons to refer terminally ill patients to a specialist were lack of time (30%), better training of specialists (23%) and end-of-life care being incompatible with other duties (19%).		
Gomez-Batiste 2017	Significant improvements were observed in the psychosocial and spiritual dimensions assessed. Patients, family members, and stakeholders all showed high levels of satisfaction. Significance of Results: This model of psychosocial care could serve as an example for other countries that wish to improve psychosocial and spiritual support. Our results confirm that specific psychosocial interventions delivered by well-trained experts can help to ease suffering and discomfort in end-of-life and palliative care patients, particularly those with high levels of pain or emotional distress.	None	Recent studies of psychological therapies have yielded relevant results in terms of regarding life meaning and wellbeing in end-of-life patients. The Palliative support teams were given the task of providing emotional support, spiritual care, and bereavement assistance for patients and their families.	Significant improvements were observed in the psychosocial and spiritual dimensions assessed. Patients, family members, and stakeholders all showed high levels of satisfaction.	This model of psychosocial care could serve as a template for other regions or countries that wish to further improve psychosocial support for this highly vulnerable and and often underserved population. More research is needed to develop geater standardization to ensure the replicability and consistency of such programs.
Gratz 2017	Spiritual care training for volunteers should cover the following themes and practical assignments: (1) definition of central concepts of spirituality and spiritual care; (2) meaning of belief systems; (3) spiritual needs and resources; (4) personal manner and ability to relate meaningfully; (5) referral to appropriate pastoral care/chaplains/spiritual advisors; (6) rituals and creativity in spiritual care; (7) voicing and acknowledging own spirituality; (8) facing and initiating spiritual encounters. Course aims were identified concerning knowledge, skills, and attitude.	None. Course aim is the concept of spirituality and the differences between spirituality, belief and religiosity. The role of spirituality in the context of taking stock of life, search for meaning, meaning of illness and values.	Knowledge: definitions of spirituality and spiritual care, meanings- and belief system, spiritual needs and resources. Skills: personal manner and ability to relate meaningfully, referral to chaplains. Attitudes: Voicing and acknowledging own spirituality, facing and initiating spiritual encounters.	Spiritual care training for volunteers should cover the following themes and practical assignments: (1) definition of central concepts of spirituality and spiritual care; (2) meaning of belief systems; (3) spiritual needs and resources; (4) personal manner and ability to relate meaningfully; (5) referral to appropriate pastoral care/chaplains/spiritual advisors; (6) rituals and creativity in spiritual care; (7) voicing and acknowledging own spirituality; (8) facing and initiating spiritual encounters. Course aims were identified concerning knowledge, skills, and	Hospice volunteers' spiritual care training has to be considered against the background of their role and motivation, which vary widely from country to country. The course aims should meet the benefits of a competencies-based approach as "competencies provide clrity regarding what a healthcare professional from a particular discipline needs to know to perform their role consistently and effectively.
Kisvetrova 2017	The least frequently implemented activity by RNs was "Show the patient's willingness to discuss death" and the most frequent activity was "Threat to the patient's dignity and respect." The highest utilization rate of nursing activities was reported in the physical dimension, while the lowest utilization rate of nursing activities was in the social dimension set. Significant predictors for the high utilization rate of physical dimension set activities were hospice care departments, long-term care facilities (LTCFs), and the age of RNs. Hospice departments were also a predictor of high utilization rate of activities in the psychological, spiritual, and social dimension set activities. Conclusions: With the exception of hospice departments, RNs used activities encouraging psychological, spiritual, and social comfort for end-of-life patients less frequently than the physical dimension.	INTRODUCTION: Spiritual comfort needs pertain to meaning in one's life, and one's understood relationship with a higher being. METHOD / RESULTS: assessed are the following spiritual dimensions: a) treat individual with dignity and respect, b) provide privacy and quiet times for spiritual activities, c) arrange visit by individual's spiritual advisors, d) facilitate obtaining spiritual support for patient and family, e) communicate willingness to discuss death	Hospice departments were also a predictor of high utilization rate of activities in the psychological, spiritual, and social dimension set activities.		
Loeffen 2017	The final IPPCP comprised five domains: (1) IPPCP data, (2) basics, (3) social, (4) psychosocial and spiritual and (5) physical care. Each domain covered various components. In both pilots, the IPPCP was considered a comprehensive document that covered all areas of paediatric palliative care and was experienced as an improvement to the present situation. However, the current form was regarded to lack user-friendliness. Conclusion We propose a set of essential components of a comprehensive IPPCP for paediatric palliative care with extra attention for advance care planning and anticipatory action. Patients' and parents' preferences and desires are included next to the recommendations of the evidence-based guideline 'Palliative care for children'.	None	The final IPPCP comprised five domains: (1) IPPCP data, (2) basics, (3) social, (4) psychosocial and spiritual and (5) physical care. Each domain covered various components.		

Macpherson 2017	Methods to address and overcome inherent ethical difficulties and reveal relational practice were developed. Data included naturally occurring conversations between practitioners relating to one family, systematically exploring difficulties faced and meaning constructed in depth. The data were then used to fictionalise a family account that re-presented actual challenges practitioners confronted. Reflexivity was used to unfold the layers of complex influences and ethical issues practitioners face when grappling with making meaning. Even with a clear understanding of processes and willingness to facilitate difficult conversations, practitioners face tensions between respect for a dying patient's needs, avoiding undermining the family culture and meeting children's needs. Contrary to the requirement to practise from an evidence base, some situations require the ability to work with 'not knowing'. Limitations include the subjective nature of the account and the smoothing over of complexity pertaining to lived experience.	None	Even with a clear understanding of processes and willingness to facilitate difficult conversations, practitioners face tensions between respect for a dying patient's needs, avoiding undermining the family culture and meeting children's needs.		
Olsson 2017	The living arrangements differed between younger and older participants; however, the lossrelated variables did not differ. Significant positive changes were found regarding a sense of meaning in their future life and life satisfaction. The helpfulness of the group was assessed as high/very high and the group brought a valuable fellowship with others in a similar situation. Universality and beneficial interactions were reported and strengthened psychosocial well-being developed over time. This change, according to the young people themselves, may be attributed to the group support. The findings are useful for planning interventions to support young people in bereavement in order to enhance their psychosocial well-being.	None	No mention of spiritualcare. With the group exerience and during the first year of bereavement, their sense of meaning in future life, feelings of loneliness, and satisfaction with most life domains changed positively. About 14 months after their loss, the young individuals still felt 'different' than their peers and not always understood by others due to parental bereavement.	Significant positive changes were found regarding a sense of meaning in their future life and life satisfaction. The helpfulness of the group was assessed as high/very high and the group brought a valuable fellowship with others in a similar situation. Universality and beneficial interactions were reported and strengthened psychosocial well-being developed over time. This change, according to the young people themselves, may be attributed to the group support.	
Paal 2017	The results demonstrate that taking a spiritual history is a complex and challenging task, requiring a number of personal qualities of the interviewer, such as 'being present', 'not only hearing, but listening', 'understanding the message beyond the words uttered', and 'picking up the words to respond'. To 'establish a link of sharing', the interviewer is expected 'to go beyond the ethical stance of neutrality'. The latter may cause several dilemmas, such as 'fear of causing more problems', 'not daring to take it further', and above all, 'being ambivalent about one's role'. Interviewer has to be careful in terms of the 'patient's vulnerability'. To avoid causing harm, it is essential to propose 'a follow-up contract' that allows responding to 'patient's yearning for genuine care'. These findings combined with available literature suggest that the quality of spiritual history taking will remain poor unless the health-care professionals revise the meaning of spirituality and the art of caring on individual level.	INTRODUCTION: The semi-structured spiritual history tool F (faith and belief) I (importance) C (community) A (address in care) has been introduced and tested as forthcoming in clinical practice |This indicates that to attend and address spirituality, health-care professionals should be guided and encouraged to shift from medical, technical, and clinical to the ''whole way of being in the world'' for their personal lives	The results demonstrate that taking a spiritual history is a complex and challenging task, requiring a number of personal qualities of the interviewer, such as 'being present', 'not only hearing, but listening', 'understanding the message beyond the words uttered', and 'picking up the words to respond'. To 'establish a link of sharing', the interviewer is expected 'to go beyond the ethical stance of neutrality'. The latter may cause several dilemmas, such as 'fear of causing more problems', 'not daring to take it further', and above all, 'being ambivalent about one's role'. Interviewer has to be careful in terms of the 'patient's vulnerability'. To avoid causing harm, it is essential to propose 'a follow-up contract' that allows responding to 'patient's yearning for genuine care'.		
Shaw 2017	Open questions about the patients' experiences, feelings or understanding in the context of talk about their troubles, were found to regularly elicit talk concerning end-of-life. These questions were designed in ways that invite patients to discuss troubling aspects of their cancer journey, without making discussion of this topic an interactional requirement. That is, the interactional work required to not engage in such talk is minimised. This choice is provided through the open question design, the degree to which negative feeling descriptors are specified, and the sequential context of the question. Conclusion: The analysis shows that therapists provide patients with the opportunity to talk about end-of-life in a way that is supportive of the therapeutic relationship. The readiness of patients to engage in end-of-life talk displays the salience of this topic, as well as the reflective space provided by CALM therapy.	INTRODUCTION: The CALM intervention is an attachment- based supportive-expressive therapy with specific attention to four domains 1) managing symptoms and navigating the health care system; 2) understanding how the disease has changed self and relations with close others; 3) spiritual meaning and purpose; and 4) future, hope and mortality.	Open questions about the patients' experiences, feelings or understanding in the context of talk about their troubles, were found to regularly elicit talk concerning end-of-life. These questions were designed in ways that invite patients to discuss troubling aspects of their cancer journey, without making discussion of this topic an interactional requirement. That is, the interactional work required to not engage in such talk is minimised. This choice is provided through the open question design, the degree to which negative feeling descriptors are specified, and the sequential context of the question. [EXCLUDE: PSYCHOSOCIAL CARE?]		

Toivonen 2017	Supporting the spirituality of older people with dementia was seen as understanding their spirituality within a framework of person-centeredness and individuality. The participants came to understand the spiritual needs of older people with dementia through both verbal and nonverbal expression and by learning about older people's individual spiritual backgrounds. Meeting spiritual needs meant approaching the person with dementia as a valuable human as well as paying attention, to and supporting, his/her personal philosophy of life within nursing care. Conclusion: Learning and developing an understanding of the spiritual needs of older people with dementia is challenging. The nurses offered person-centred, spiritual care, to people with dementia from a variety of perspectives, which is important in the provision of comprehensive care. There is a need to find usable tools to help nurses to learn and understand the individual spiritual needs of older people with dementia and to explore how these older adults experience having their spirituality supported within their nursing care.	INTRODUCTION: Spirituality is defined as a search for answers to existential questions about the meaning of life and the individual's relationship with the sacred or transcendent.	Supporting the spirituality of older people with dementia was seen as understanding their spirituality within a framework of person-centeredness and individuality. The participants came to understand the spiritual needs of older people with dementia through both verbal and nonverbal expression and by learning about older people's individual spiritual backgrounds. Meeting spiritual needs meant approaching the person with dementia as a valuable human as well as paying attention, to and supporting, his/her personal philosophy of life within nursing care.		
Van de Geer 2017a (Training hospital staff..)	All 85 patients had high scores on spiritual themes and involvement. Patients reported that attention to their spiritual needs was very important. We found a significant (p = 0.008) effect on healthcare professionals' attention to patients' spiritual and existential needs and a significant (p = 0.020) effect in favour of patients' sleep. No effect on the spiritual distress of patients or their proxies was found.	Based on the consensus definition by the Taskforce Spiritual Care of the EAPC and the multidisciplinary Dutch guideline spiritual care in palliative care.	Patients reported that attention to their spiritual needs was very important.	We found a significant (p = 0.008) effect on healthcare professionals' attention to patients' spiritual and existential needs and a significant (p = 0.020) effect in favour of patients' sleep. No effect on the spiritual distress of patients or their proxies was found.	The cliical effects of spiritual care training for health care professionals can be measured using patient-reported outcomes.The SAIL was not developed t measure short-term or longterm effects in clinical practice, it contains themes that palliatieve patients find important: meaningfullness, trust, acceptance, caring for others and connectedness with nature.
Van de Geer 2017b (Multidisciplinary Training)	For nurses (n = 214), 7 of 8 barriers to SC were decreased after 1 month, but only 2 were still after 6 months. For physicians (n = 41), the training had no effect on the barriers to SC. Nurses improved in 4 of 6 competencies after both 1 and 6 months. Physicians improved in 3 of 6 competencies after 1 month but in only 1 competency after 6 months. Significance of Results: Concise SC training programs for clinical teams can effect quality of care, by improving hospital staff competencies and decreasing the barriers they perceive. Differences in the effects of the SC training on nurses and physicians show the need for further research on physicians' educational needs on SC.	Based on the multidisciplinary Dutch guideline spiritual care in palliative care	Core skills were screening/assessng spiritual needs, counselling patiens (matching their owm profssional role), and referring patients to specialists when the patients are in a crisis.	For nurses (n = 214), 7 of 8 barriers to SC were decreased after 1 month, but only 2 were still after 6 months. For physicians (n = 41), the training had no effect on the barriers to SC. Nurses improved in 4 of 6 competencies after both 1 and 6 months. Physicians improved in 3 of 6 competencies after 1 month but in only 1 competency after 6 months.	Measurig effects based on self-assessment tools (SAIL) does not give a clear picure of health car eprofessionals. We consider the fact that this training was performed with health care professionals on regular wards, to be a strength. Also, a training of 90-180 min for hospital staff and health-care professional-reported outcomes.In nursing homes, the results were maintained after 6 months. Multdicdisciplinary education was mandatory.
Van de Geer - 2017c (improving spiritual care)	During preintervention interviews, chaplains describe the baseline situation of palliative care in Dutch hospitals, barriers, and opportunities for improving spiritual care. In the postintervention interviews, characteristics of the training, effects, and critical success factors were identified. Positive effects such as lowering barriers, increasing health care professionals' competences, and increasing health care chaplains' profile are possible. Chaplain-led, multidisciplinary spiritual care training is a feasible method to start implementation of spiritual care in hospitals, as described in the multidisciplinary guideline.	Based on the consensus definition by the Consensus Conference in the US, the Taskforce Spiritual Care of the EAPC and the multidisciplinary Dutch guideline spiritual care in palliative care.	According to chaplains, raining health care professionals in SC as an implementation strategy for the SC guideline was a fruitfull endeavor.	Positive effects such as lowering barriers, increasing health care professionals' competences, and increasing health care chaplains' profile are possible. Chaplain-led, multidisciplinary spiritual care training is a feasible method to start implementation of spiritual care in hospitals, as described in the multidisciplinary guideline.	Most relevant to an exploration for an implementation strategy: 1) the context of PC in hospitals, use of a diagnostic tool , 2) the chaplain's views on developing spiritual care, research based chaplaincy, and c) new experience and knowledge based on participation in the study, e.g. organizational structures an dnew relations with physicians and managers. For project-based improvement of SC in hospitals using implementation of a guideline, a clear mandate and ownership departments bottom-up commitment, chaplains attitudes of authenticity, and personal commitment to the team are critical success factors. Presenting SCas connected to health care, and time.

Walker 2017	The study shows that the advantages of a broader definition of spirituality lie in ‘‘spiritual care’’ no longer being bound to one single profession, namely that of the chaplain. It also opens the way for nurses and volunteers—irrespective of their own religious beliefs—to provide spiritual end-of-life care to patients in hospices. If the hospice nurses and volunteers were able to mitigate the patients’ fear not only by using medications but also in a psychosocial or spiritual respect, then they saw this as a successful psychological and spiritual guidance. The spiritual guidance is to some degree independent of religious belief because it refers to a ‘‘spirit’’ or ‘‘inner core’’ of human beings. But this guidance needs assistance from professional knowledge considering religious rituals if the patients are deeply rooted in a (non-Christian) religion. Here, the lack of knowledge could be eliminated by further education as an essential but not sufficient condition.	INTRODUCTION: The concept of spirituality by Cicely Saunders, based on the work of Victor Frankl: ‘[W]e can always persevere with the practical. Care for the physical needs; the time taken to elucidate a symptom, the quiet acceptance of family’s angry demands, the way nursing care is given, can carry at all and can reach the most hidden places. Though this may be all we can offer to inarticulate spiritual pain, it may be enough as our patients finally face the truth on the other side of death. || At a conference in the year 2009, where ‘‘over forty US leaders in palliative care, as well as spirituality and theology’’ were present, spirituality was defined as ‘‘the aspect of humanity that refers to the way individuals seek and express meaning and purpose and the way they experience their connectedness to the moment, to self, to others, to nature, and to the significant or sacred’’ (Puchalski 2010). This definition conceives spirituality as a broader concept than religion. Spirituality can have to do with beauty, values, altruism, idealism and ‘‘awareness of the tragic’’ (Cohen et al. 2012, pp. 801–802).	The study shows that the advantages of a broader definition of spirituality lie in ‘‘spiritual care’’ no longer being bound to one single profession, namely that of the chaplain. It also opens the way for nurses and volunteers—irrespective of their own religious beliefs—to provide spiritual end-of-life care to patients in hospices. If the hospice nurses and volunteers were able to mitigate the patients’ fear not only by using medications but also in a psychosocial or spiritual respect, then they saw this as a successful psychological and spiritual guidance. The spiritual guidance is to some degree independent of religious belief because it refers to a ‘‘spirit’’ or ‘‘inner core’’ of human beings. But this guidance needs assistance from professional knowledge considering religious rituals if the patients are deeply rooted in a (non-Christian) religion.	The concept of spirituality as something that includes God, but also exceeds religion, 'everybody is spiritual whether they are aware of the fact or not' . Hospices usually have a room of tranquility or a chapel. Give the room a spiritual character. Room for religious bliefs and diversity. Spirituality as an attitude (spirit of compassion), Supporting spiritual practices, alone or together, sometims it is enough that someone is 'just there'.	The spiritual guidance is to some degree independent of religious belief because it refers to a ‘‘spirit’’ or ‘‘inner core’’ of human beings. But this guidance needs assistance from professional knowledge considering religious rituals if the patients are deeply rooted in a (non-Christian) religion. Here, the lack of knowledge could be eliminated by further education as an essential but not sufficient condition.
Werner	The doctor communicated in a recognising manner, expressing respect for the patient as a subject and an authority of his own experiences. The doctor and patient succeeded in creating a good working alliance characterised by warmth and trust. Within this context, there was room for the doctor to challenge the patient’s views and communicate disagreement. Conclusions: The doctor succeeds in conveying and maintaining hope. Within a good working alliance with the patient the doctor can convey hope by balancing between supporting and challenging him. Exploring and grasping the patient’s real concerns is essential for being able to relieve and comfort him and convey hope.	None	The doctor communicated in a recognising manner, expressing respect for the patient as a subject and an authority of his own experiences. The doctor and patient succeeded in creating a good working alliance characterised by warmth and trust. Within this context, there was room for the doctor to challenge the patient’s views and communicate disagreement.		

1) For the full references, see Table 1 and Manuscript
